# PI3Kγ promotes obesity-associated hepatocellular carcinoma by regulating metabolism and inflammation

**DOI:** 10.1016/j.jhepr.2021.100359

**Published:** 2021-09-02

**Authors:** Barbara Becattini, Ludovic Breasson, Claudia Sardi, Fabio Zani, Giovanni Solinas

**Affiliations:** 1 The Wallenberg Laboratory, Department of Molecular and Clinical Medicine at Institute of Medicine, University of Gothenburg, Sahlgrenska University Hospital, Gothenburg, Sweden; 2 The Francis Crick Institute, London, UK

**Keywords:** NAFLD, NASH, Insulin, AKT, mTOR, AST, aspartate aminotransferase, BMDM, bone marrow-derived macrophages, DEN, diethylnitrosamine, GTT, glucose tolerance test, ITT, insulin tolerance test, HCC, hepatocellular carcinoma, HFD, high-fat diet, PI3K, phosphatidylinositides-3 kinase, PTEN, phosphatase and tensin homolog, RT, room temperature, TUNEL, terminal deoxynucleotidyl transferase dUTP nick-end labelling, WT, wild-type

## Abstract

**Background & Aims:**

Phosphatidylinositides-3 kinases (PI3Ks) are promising drug targets for cancer therapy, but blockage of PI3K-AKT signalling causes hyperglycaemia, hyperinsulinaemia, and liver damage in patients, and hepatocellular carcinoma (HCC) in mice. There are 4 PI3Ks: PI3Kα, PI3Kβ, PI3Kδ, and PI3Kγ. The role of PI3Kγ in HCC is unknown.

**Methods:**

We performed histopathological, metabolic, and molecular phenotyping of mice with genetic ablation of PI3Kγ using models where HCC was initiated by the carcinogen diethylnitrosamine (DEN) and promoted by dietary or genetic obesity (ob/ob). The role of PI3Kγ in leucocytes was investigated in mice lacking PI3Kγ in haematopoietic and endothelial cells.

**Results:**

Loss of PI3Kγ had no effects on the development of DEN-induced HCC in lean mice. However, in mice injected with DEN and placed on an obesogenic diet, PI3Kγ ablation reduced tumour growth, which was associated with reduced insulinaemia, steatosis, and expression of inflammatory cytokines. ob/ob mice lacking PI3Kγ, and mice with diet-induced obesity lacking PI3Kγ in leucocytes and endothelial cells did not display improved insulin sensitivity, steatosis, metabolic inflammation, or reduced tumour growth. However, these mice showed a reduced number of tumours, reduced liver infiltration by neutrophils, and reduced hepatocyte proliferation acutely induced by DEN.

**Conclusions:**

Loss of PI3Kγ reduces tumour development in obesity-promoted HCC through multiple cell types and mechanisms that include improved insulinaemia, steatosis, and metabolic inflammation as well as the regulation of acute neutrophil infiltration and compensatory hepatocyte proliferation. PI3Kγ-selective inhibition may represent a novel therapeutic approach to reduce HCC initiation and slow HCC progression.

**Lay summary:**

Class-1 phosphatidylinositides-3 kinases (PI3Ks) are critical targets in cancer therapy, but complete inhibition of all isoforms causes liver damage, hyperglycaemia, and insulinaemia. Here we show that selective ablation of the PI3Kγ isoform dampens tumour initiation and growth in a mouse model of carcinogen-initiated and obesity-promoted hepatocellular carcinoma (HCC). The effect of PI3Kγ ablation on reduced tumour growth was explained by reduced tumour cell proliferation, which was associated with reduced insulin levels, liver lipids, and reduced expression of tumour-promoting cytokines. PI3Kγ ablation in leucocytes of obese mice had no effects on tumour size. However, it reduced tumour number in association with reduced carcinogen-induced neutrophil infiltration and hepatocyte proliferation in livers of obese mice. Inhibition of PI3Kγ may thus reduce HCC initiation and growth in obese subjects by a mechanism involving reduced metabolic stress and insulinaemia and reduced carcinogen-induced neutrophil infiltration to the fatty liver.

## Highlights

• PI3Kγ ablation does not affect carcinogen-induced liver cancer in lean mice.
• PI3Kγ ablation reduces carcinogen-induced liver cancer in obese mice.
• Systemic PI3Kγ ablation reduces hyperinsulinaemia, steatosis, metabolic inflammation, and growth of liver tumours.
• PI3Kγ ablation in leucocytes and endothelial cells reduces neutrophil infiltration and hepatocyte proliferation acutely induced by carcinogen in the fatty liver.

## Graphical abstract

**Figure:**
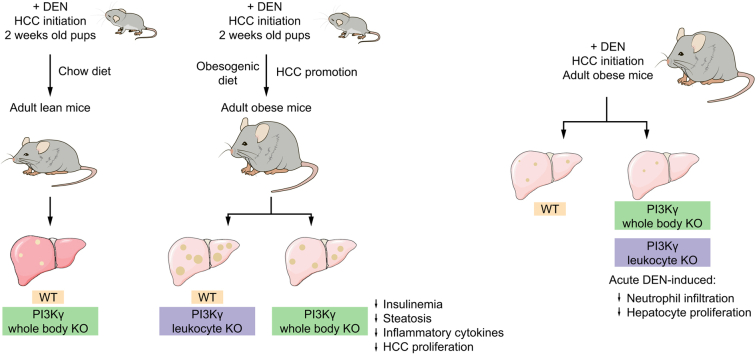

## Introduction

Hepatocellular carcinoma (HCC) cells display aberrant insulin signalling,^1^^,^^2^ and obesity and hyperinsulinaemia are associated with increased HCC risk.^3^^,^^4^ The class-1 phosphatidylinositides-3 kinases (PI3Ks) are a family of lipid kinases comprising 4 catalytic subunits: PI3Kα, PI3Kβ, PI3Kδ, and PI3Kγ,^5^ with activity which is essential for growth factors signalling and the metabolic action of insulin.^5^ Aberrant PI3K signalling is a frequent alteration driving tumour progression.^6^ Sustained PI3K activation in the liver by ablation of the phosphatase and tensin homolog (PTEN) in mice^7^ or by transplantation of pancreatic islets into the livers of diabetic rats causes HCC.^8^ An obstacle to the development of PI3K-targeted HCC therapy is liver damage, as transaminitis is frequently observed in patients treated with pan-PI3K inhibitors.^9^ Complete blockage of PI3K-AKT signalling causes hyperinsulinaemia and liver damage promoting HCC in mice.^10^

Another roadblock to the development of PI3K-targeted therapies is the role of PI3K in the metabolic action of insulin.^11^ Inhibition of all PI3K isoforms causes hyperglycaemia and hyperinsulinaemia, which activates PI3K in the tumour, dampening the therapeutic index of pan-PI3K inhibitors.^11^ Circumventing hyperglycaemia and hyperinsulinaemia caused by PI3K inhibition is thus fundamental to harness the full potential of PI3K-targeted cancer therapy.^11^

Insulin signalling in the hepatocyte controlling glucose homeostasis is mediated by redundant PI3Kα and PI3Kβ activities, whereas PI3Kδ and PI3Kγ are largely dispensable for insulin action.^12^ Hence, specific isoform-selective PI3K inhibitors may be effective in the treatment of HCC. Indeed, selective ablation of PI3Kγ protects mice from high-fat diet (HFD)-induced obesity, fatty liver, metabolic inflammation, and insulin resistance.[13], [14], [15], [16], [17] Thus, targeting PI3Kγ may interfere with systemic mechanisms by which obesity promotes HCC.

Here we have investigated the role of PI3Kγ in mouse models of diethylnitrosamine (DEN)-initiated and obesity-promoted HCC.

## Materials and methods

### *In vivo*

Mice were males C57BL/6J. Experiments were authorised by the Veterinary committee of canton Fribourg and the Research Animal Ethics Committee of the University of Gothenburg. PI3Kγ^-/-^ mice, ob/ob-PI3Kγ^-/-^ mice, and PI3Kγ^HE^ mice were described.^13^^,^^14^

**Fig. 1:**
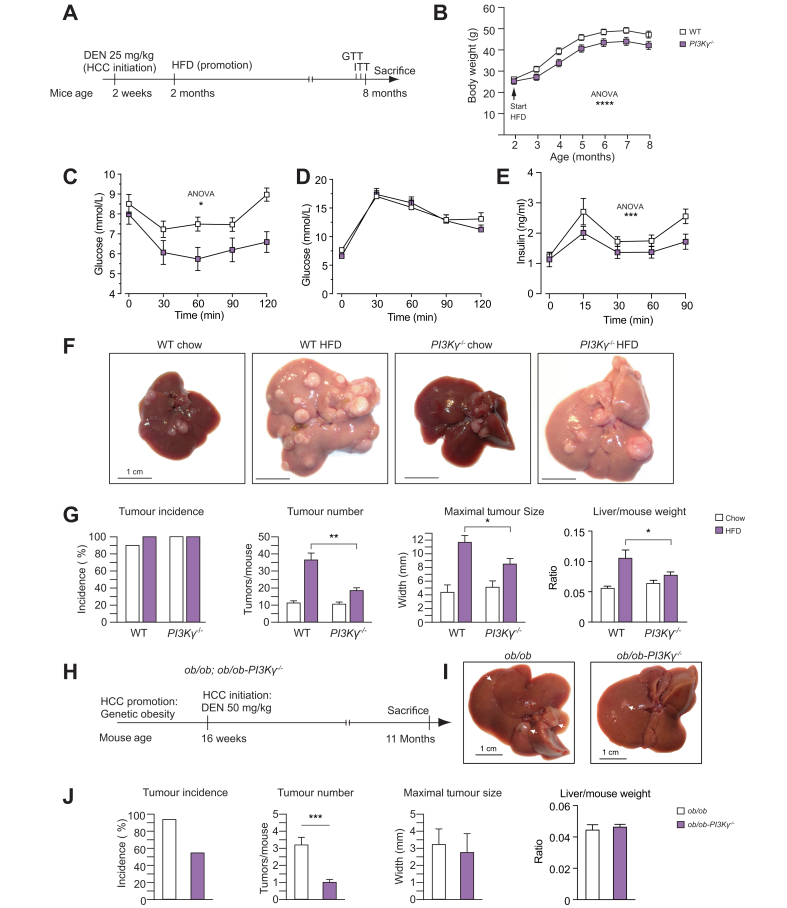
PI3Kγ ablation protects mice from obesity-mediated promotion of HCC. (A) Experimental time-course for the model of diet-induced obesity promotion of the growth of DEN-initiated HCC. (B) Growth curves of WT and PI3Kγ^-/-^ mice placed on a HFD as described in (A). (C) Insulin tolerance test of the mice in B at 7 months and 3 weeks of age. (D) GTT of the mice in (B) at the age of 7 months and 2 weeks. (E) Serum insulin levels during GTT of the mice in (B). (F) Representative images of livers from 8-month-old WT and PI3Kγ^-/-^ mice treated as described in (A). (G) Tumour incidence, tumour number, maximal tumour size, and liver weight/mouse weight ratio of the WT and PI3Kγ^-/-^ mice described above. (H) Experimental time-course for the obesity promotion of DEN-driven HCC initiation in *ob/ob* and *ob/ob-*PI3Kγ^-/-^ mice kept on a chow diet. (I) Representative images of livers from 11-month-old *ob/ob* and *ob/ob-*PI3Kγ^-/-^ mice treated as indicated in (H). (J) Tumour incidence, tumour number, maximal tumour size, and liver weight/mouse weight ratio of the *ob/ob* and *ob/ob-*PI3Kγ^-/-^ mice described in (H). n = 12 mice per group for WT and PI3Kγ^-/-^ mice placed on a HFD; n = 5–10 mice per group for WT and PI3Kγ^-/-^ mice kept on chow diet; and n = 14 mice per group for *ob/ob* and *ob/ob-*PI3Kγ^-/-^ mice. 2-way ANOVA in (B-E), Mann-Whitney test in (G, J). ∗*p* <0.05, ∗∗*p* <0.01, ∗∗∗*p* <0.001, ∗∗∗∗*p* <0.0001. Data are expressed as means, and error bars indicate standard errors. DEN, diethylnitrosamine; GTT, glucose tolerance test; HCC, hepatocellular carcinoma; HFD, high-fat diet; PI3Kγ, phosphatidylinositides-3 kinase-gamma; WT, wild-type.

For the model of obesity promotion of HCC growth (Fig. 1, Fig. 5A), 25 mg/kg DEN were injected into 2-week-old mice. At the age of 2 months, mice were fed either a chow or HFD (F3282, Bio-Serv, Rostock, Germany) until 8 months of age. For the model of obesity promotion of HCC initiation, 16-week-old *ob/ob-PI3K*γ^-/-^ mice and control *ob/ob* mice; or 16-week-old diet-induced obese PI3Kγ^HE^ mice and PI3Kγ^F/F^ mice were injected with DEN as indicated (Fig. 1, Fig. 5E). For glucose and insulin tolerance tests, mice were fasted for 4 h and injected with either 1 g/kg body weight of glucose, or 1 IU insulin/kg. Glycaemia was measured using a glucometer.

### Histology

Tissues were formalin-fixed and paraffin-embedded. Neutrophil (CD11b^+^Ly6G^+^ cells) were stained as described,^13^^,^^18^ and counted in 3 random fields per section.

For Mac2, F4-80, and Ki67 staining, after blocking with 3% of H_2_O_2_ for 20 min at room temperature (RT), and horse serum 1:75 in PBS, 20 min RT; sections were incubated overnight at 4°C with anti-MAC2 (Cedarlane, Burlington, Ontario, Canada), -F4-80 (Biorad, Hercules, CA, United States) or -Ki67 (Cell Signaling, Danvers, MA, United States) in PBS. Tissue sections were then washed and incubated with IgG biotinylated antibody for 30 min at RT, washed in PBS and incubated in ABC reagent (Vector Laboratories, Burlingame, CA, United States) for 60 min at RT, PBS washed, incubated with 3,3′-diaminobenzidine as substrate (Sigma); and haematoxylin counterstained. Positive cells were counted in 3 fields per section. Terminal deoxynucleotidyl transferase dUTP nick-end labelling (TUNEL) was performed according to the manufacturer (TMR Red, Roche, Basel, Switzerland).

### Molecular analysis

Real-time PCR was performed using cyclophilin as internal control (Table S1) as described.^13^ For immunoblotting, frozen liver was lysed in 20 mM Tris-HCl, 5% glycerol, 138 mM NaCl, 2.7 mM KCl, 1% NP-40, 5 mM EDTA with phosphatases and proteases inhibitors. Protein extracts were resolved by SDS-PAGE, and transferred onto polyvinylidene fluoride membrane. Primary antibodies were from Cell Signaling except PI3Kγ (Wymann Lab) and PI3Kδ (Millipore, Burlington, MA, United States). Serum insulin was measured using ELISA (Crystal Chem, Elk Grove Village, IL, United States). Liver triglycerides and serum aspartate aminotransferase (AST) levels were measured using commercial assays (Abcam, Cambridge, United Kingdom).

### Statistical analysis

Sample size was determined empirically basing on previous experience.^19^ Data are expressed as means and error bars. The *p* values were calculated using either Wilcoxon–Mann-Whitney *U* test or Student *t* test for simple comparison and 2-way ANOVA followed by Sidak when 2 different variables were considered. A value of *p* <0.05 was considered statistically significant. Statistical analysis was performed with GraphPad Prism (GraphPad Software Inc., San Diego, CA, USA).

## Results

### PI3Kγ ablation reduces HCC in DEN-injected mice made obese by a HFD

We investigated mice lacking a functional PI3Kγ protein (*PI3K*γ^-/-^) in 2 different models of obesity-mediated promotion of HCC.^19^ The first model was designed to study the effects of obesity on HCC growth. The second model investigated the effects of obesity on tumour initiation by the carcinogen. In the first model (Fig. 1A), 2-week-old mice received 25 mg of DEN per kg of body weight, and at the age of 2 months, were either kept on a chow diet or placed on an obesogenic HFD until the age of 8 months, when the mice were sacrificed. As expected, *PI3K*γ^-/-^ mice placed on a HFD gained less weight than wild-type (*WT*) mice[13], [14], [15]^,^^17^ (Fig. 1B). Compared with *WT* mice, *PI3K*γ^-/-^ mice displayed a sustained improvement in insulin sensitivity, and although glucose tolerance was similar between genotypes, *PI3K*γ^-/-^ mice showed reduced insulin levels during the glucose tolerance test (Fig. 1C–E). Except for one *WT* mouse, all the mice developed HCC by the age of 8 months, and when kept on a chow diet, there was no effect of PI3Kγ ablation on tumour number and maximal tumour size (Fig. 1F,G). Gene expression profiling by real-time PCR of RNA from livers of *WT* and *PI3K*γ^-/-^ mice kept on a chow diet showed similar abundances of the macrophage marker F4/80, the cytotoxic T-cell marker CD8, and inflammation markers (Fig. S1).

*WT* mice placed on a HFD displayed a more than threefold increase in tumour number and about a 3-fold increase in maximal tumour size (Fig. 1F,G). However, these effects of HFD-feeding on tumour growth were blunted in mice lacking PI3Kγ. Overall, PI3Kγ ablation did not affect DEN-driven carcinogenesis in lean mice but reduced insulin levels and HCC progression in obese mice.

### PI3Kγ ablation reduces HCC number in DEN-injected ob/ob mice

To learn about the role of PI3Kγ on HCC initiation by DEN in obese mice, we injected 16-week-old genetically obese *ob/ob* mice and *ob/ob*-*PI3K*γ^-/-^ mice with 50 mg/kg of DEN, a dose of carcinogen not sufficient to cause HCC in adult mice unless given in association with a tumour promoter such as obesity (Fig. 1H)^19^ *ob/ob* mice and *ob/ob*-*PI3K*γ^-/-^ mice have been previously characterised,^13^ and by the age of 16 weeks (the initiation time-point), these mice display similar insulin sensitivity, liver steatosis, and metabolic inflammation.^13^ By the age of 11 months, all the *ob/ob* mice developed HCC, whereas hepatic carcinomas were observed in only half of *ob/ob*-*PI3K*γ^-/-^ mice (Fig. 1I,J). The average number of tumours per mouse was also reduced in *ob/ob-PI3K*γ^-/-^ mice, although maximal tumour size was not affected (Fig. 1I,J). PI3Kγ ablation had marginal effects on the body weight of *ob/ob* mice, and by the age of 11 months, ob/ob mice and *ob/ob-PI3K*γ^-/-^ mice showed similar body weight and hepatic steatosis (Fig. S2). It is concluded that loss of PI3Kγ reduced tumour incidence and number but not size in *ob/ob* mice injected with DEN.

### PI3Kγ activity supports proliferation in HCC of obese mice

**Fig. 2:**
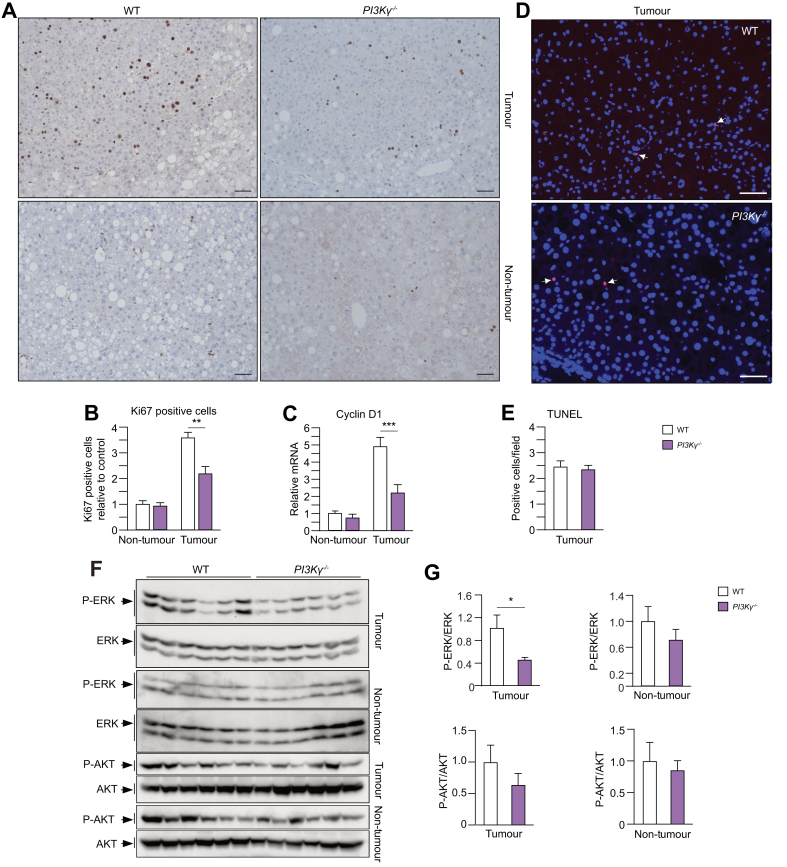
PI3Kγ ablation reduces liver tumour proliferation in obese mice. (A) Ki67 staining of liver sections from WT and PI3Kγ^-/-^ mice after DEN initiation and HFD HCC promotion as described in Fig. 1A (scale bar = 100 μm). (B) Quantification of the Ki67-positive cells in the normal livers and tumours shown above. (C) mRNA abundance of cyclin D1 in normal livers and tumours from WT and PI3Kγ^-/-^ mice described above. (D) TUNEL assay of paraffin-embedded sections of HCC from WT and PI3Kγ^-/-^ mice (scale bar = 100 μm). (E) Quantification of the number of TUNEL positive cells in (D). (F) Immunoblot analysis of protein extracts obtained from non-tumour liver and HCC of 8-month-old WT and PI3Kγ^-/-^ mice made obese by HFD-feeding as described in Fig. 1A. (G) Quantifications of the immunoblots in (F). n = 5–8 mice per group in (A,B), n = 8–10 per group in (C), n = 6 per group in (D,E), and n = 6 per group in (F,G). Mann-Whitney test in (B, C, E, G). ∗*p* <0.05, ∗∗*p* <0.01, ∗∗∗*p* <0.001. Data are expressed as means, and error bars indicate standard errors. DEN, diethylnitrosamine; GTT, glucose tolerance test; HCC, hepatocellular carcinoma; HFD, high-fat diet; PI3Kγ, phosphatidylinositides-3 kinase-gamma; TUNEL, terminal deoxynucleotidyl transferase dUTP nick-end labelling; WT, wild-type.

To investigate the mechanisms by which PI3Kγ ablation reduces HCC growth in obese mice, we measured the proliferation of liver cells by Ki67 immunostaining of liver sections of the 8-month-old *WT* mice and *PI3K*γ^-/-^ mice kept on a HFD as described in Fig. 1A. Obese *PI3K*γ^-/-^ mice showed a reduction in the number of Ki67-positive cells specifically within tumour areas, whereas the number of Ki67-positive cells in non-tumour liver was similar to that of control mice (Fig. 2A,B). Consistently, qPCR analysis of RNA from carcinoma or non-tumour liver from *PI3K*γ^-/-^ mice or *WT* mice kept on a HFD indicates that PI3Kγ ablation reduced the abundance of cyclin D1 mRNA in HCC but not in non-tumour liver (Fig. 2C). TUNEL analysis of the liver sections described above shows that PI3Kγ ablation did not affect the number of apoptotic cells in carcinoma tissue from mice placed on a HFD (Fig. 2D,E). Immunoblot analysis indicates that loss of PI3Kγ did not reduce AKT phosphorylation in non-tumour liver and HCC (Fig. 2F,G). However, the phosphorylation of the mitogen-activated protein kinase ERK was reduced in HCC of the obese *PI3K*γ^-/-^ mice (Fig. 2F,G).

Altogether, our results indicate that in obese mice bearing HCC, loss of PI3Kγ activity does not affect apoptosis or AKT phosphorylation in tumours but reduces ERK signalling, cyclin D1 expression, and tumour cell proliferation.

### PI3Kγ ablation reduces steatosis and inflammatory signals in livers of obese mice

A study reported that obesity promotes HCC via increased expression of tumour-promoting inflammatory cytokines.^19^ Hence, we have characterised metabolic inflammation in obese mice lacking PI3Kγ. Adipose tissue from 8-month-old *PI3K*γ^-/-^ mice and *WT* mice kept on HFD showed a similar number of crown-like structures, neutrophil accumulation, and expression of inflammatory markers (Figs S3A–D and S4). Two exceptions were IL-1Ra, a marker of classical ‘M1’ macrophage activation, and MMP-9, a marker of alternative ‘M2’ macrophage activation, whose mRNA abundances were respectively reduced and elevated in adipose tissues from *PI3K*γ^-/-^ mice compared with those from *WT* mice (Fig. S4). Previous studies showed reduced adipose tissue neutrophils and a more pronounced M2 polarisation of mRNA abundances of markers of macrophage activation in the adipose tissue of 16-week-old obese mice lacking PI3Kγ.^13^^,^^18^ This is likely a result of the longer exposure to HFD (8 months *vs.* 16 weeks) and to the presence of liver tumours. Indeed, compared with *ob/ob* control mice, 11-month-old *ob/ob*-*PI3K*γ^-/-^ mice showed a marked reduction of adipose tissue neutrophils and increased mRNA abundance of the ‘M2’ markers MRC-2, Arginase-1, and MMP-9 (Figs S5A–D and S6).

**Fig. 3:**
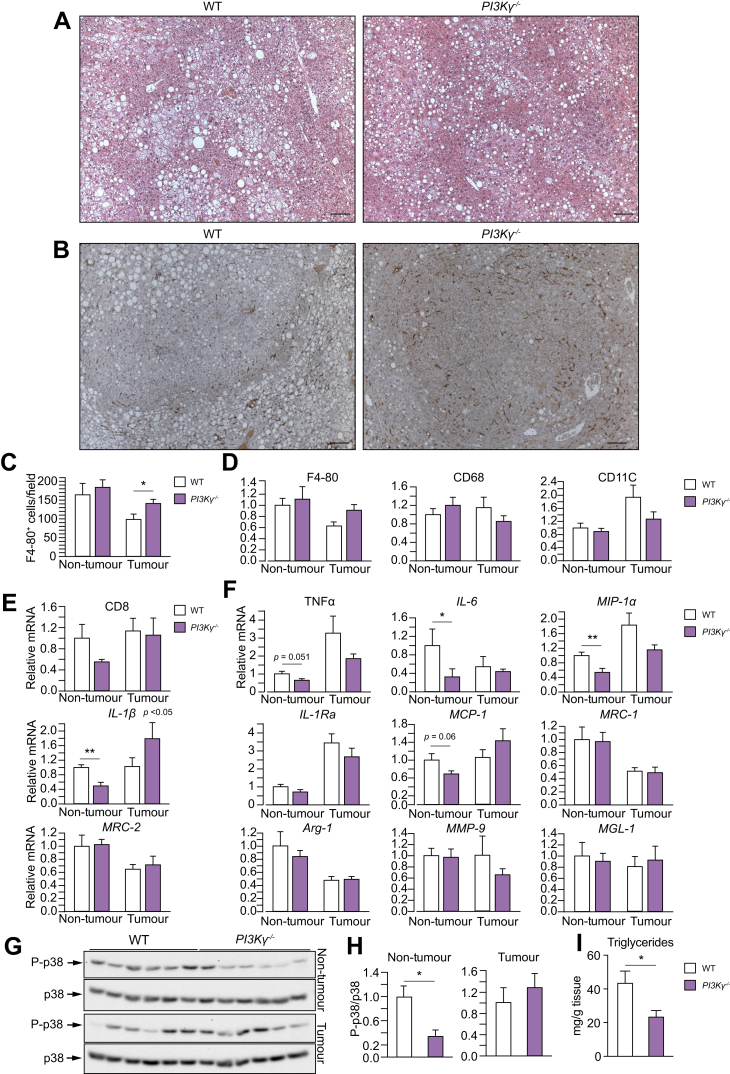
PI3Kγ ablation reduces steatohepatitis in obese mice. (A) Haematoxylin and eosin staining of liver sections from the WT and PI3Kγ^-/-^ obese mice described in Fig. 1A (scale bar = 100 μm). (B) F4/80 staining of liver sections of the WT and PI3Kγ^-/-^ obese mice above (scale bar = 100 μm). (C) Quantification of the number of F4/80-positive cells in (B). (D) mRNA abundance of general macrophage markers in normal liver and HCC from the WT and PI3Kγ^-/-^ obese mice above. (E) CD8 mRNA abundance in normal liver and HCC from the WT and PI3Kγ^-/-^ obese mice above. (F) mRNA abundance of markers of classical or alternative macrophage activation in normal liver and HCC from the WT and PI3Kγ^-/-^ obese mice above. (G) Immunoblot analysis of protein extracts obtained from non-tumour liver and HCC of 8-month-old WT and PI3Kγ^-/-^ mice made obese by HFD-feeding as described in Fig. 1A. (H) Quantifications of the immunoblots in (G). (I) Hepatic triglyceride content from the mice described above. n = 10 mice per group in (A), n = 7–8 per group in (B,C), n = 8–10 per group in (D–F), n = 6 per group in (G–I). Mann-Whitney test in (C-F, H, I). ∗*p* <0.05, ∗∗*p* <0.01. Data are expressed as means, and error bars indicate standard errors. HCC, hepatocellular carcinoma; HFD, high-fat diet; PI3Kγ, phosphatidylinositides-3 kinase-gamma; WT, wild-type.

Histology of livers from DEN-injected *WT* mice and *PI3K*γ^-/-^ mice kept on HFD showed that loss of PI3Kγ partially improved hepatic steatosis but did not reduce the accumulation of macrophages in the non-tumour liver or HCC (Fig. 3A–C). Consistently, the mRNA abundance of macrophage markers was similar in the non-tumour liver or HCC of *WT* mice and *PI3K*γ^-/-^ mice (Fig. 3D). Furthermore, the mRNA abundance of the cytotoxic T-cell marker CD8 in the non-tumour liver and HCC of *PI3K*γ^-/-^ mice was similar to that observed in *WT* mice (Fig. 3E). However, the mRNA abundances of the pro-inflammatory cytokines IL-6, MIP-1α, and IL-1β were specifically reduced in the non-tumour liver of *PI3K*γ^-/-^ mice compared with *WT* mice (Fig. 3F). Finally, phosphorylation of the stress-activated protein kinase p38 was specifically reduced in non-tumour liver from *PI3K*γ^-/-^ mice (Fig. 3G,H). Consistent with the idea of reduced steatosis, liver triglyceride content was reduced by about 50% in *PI3K*γ^-/-^ mice (Fig. 3I).

Altogether, our results indicate that, in the DEN-initiation and HFD-promotion HCC model (Fig. 1A), mice lacking PI3Kγ are partially protected from steatosis, and display reduced p38 signalling and reduced expression of pro-inflammatory cytokines in specifically non-tumour liver tissue.

### PI3Kγ ablation reduces neutrophil recruitment and hepatocyte proliferation acutely induced by DEN in livers of obese mice

**Fig. 4:**
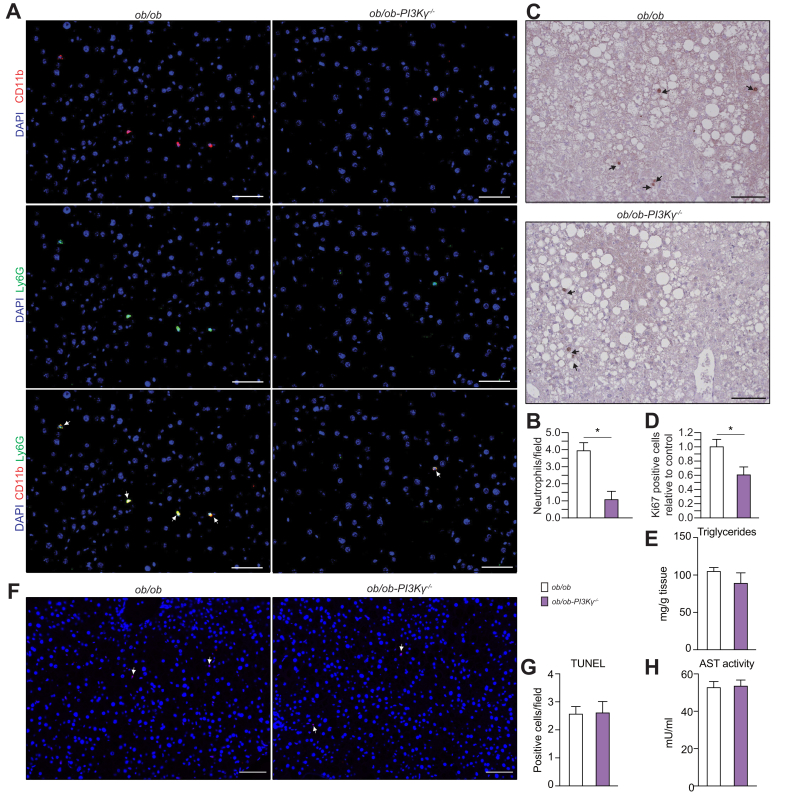
PI3Kγ drives neutrophil recruitment and hepatocyte proliferation in livers of obese mice following a DEN injection. (A) Immunostaining of neutrophils in liver sections from 16-week-old *ob/ob* and *ob/ob-*PI3Kγ^-/-^ mice collected 48 h after DEN injection. Neutrophils are defined as CD11b and Ly6G double-positive cells (scale bar = 100 μm). (B) Quantification of the number of neutrophils in (A). (C) Ki67 staining of liver sections from the mice above (scale bar = 100 μm). (D) Quantification of the number of Ki67-positive cells from (C). (E) Hepatic triglyceride content from the mice described above. (F) TUNEL staining of paraffin-embedded sections of livers from *ob/ob* and *ob/ob-*PI3Kγ^-/-^ mice (scale bar = 100 μm). (G) Quantification of the number of TUNEL positive cells in (F). (H) AST activity in serum from the mice above. n = 4–6 mice per group. Mann-Whitney test in (B, D, E, G, H). ∗*p* <0.05. Data are expressed as means, and error bars indicate standard errors. AST, aspartate aminotransferase; DEN, diethylnitrosamine; PI3Kγ, phosphatidylinositides-3 kinase-gamma; TUNEL, terminal deoxynucleotidyl transferase dUTP nick-end labelling.

Neutrophils transiently infiltrate mice livers in response to a DEN injection and exacerbate damage, which drives hepatocyte proliferation at the site of the DEN lesion, thereby promoting HCC initiation.^20^ PI3Kγ activity in neutrophils and endothelial cells plays a crucial role in neutrophil recruitment during acute inflammation^21^^,^^22^ and adult *ob/ob* mice lacking PI3Kγ injected with DEN showed reduced HCC incidence and number (Fig. 1J). Thereby, we have quantified the number of neutrophils and proliferating hepatocytes in livers collected from *ob/ob*-*PI3K*γ^-/-^ mice and *ob/ob* control mice 48 h after DEN injection. PI3Kγ ablation in obese mice caused a 4-fold reduction in the number of neutrophils recruited to their livers 48 h following the DEN injection (Fig. 4A,B) and about a 40% reduction in the number of proliferating hepatocytes (Fig. 4C,D). Liver triglyceride content and liver damage were similar in livers from *ob/ob*-*PI3K*γ^-/-^ mice and *ob/ob* mice (Fig. 4E–H).

We conclude that PI3Kγ activity is required for efficient neutrophil recruitment and hepatocyte proliferation in the fatty liver of obese mice acutely induced by a DEN injection. This effect was not associated with differences in steatosis or liver damage.

### PI3Kγ ablation in haematopoietic and endothelial cells of obese mice reduces HCC initiation but not growth

**Fig. 5:**
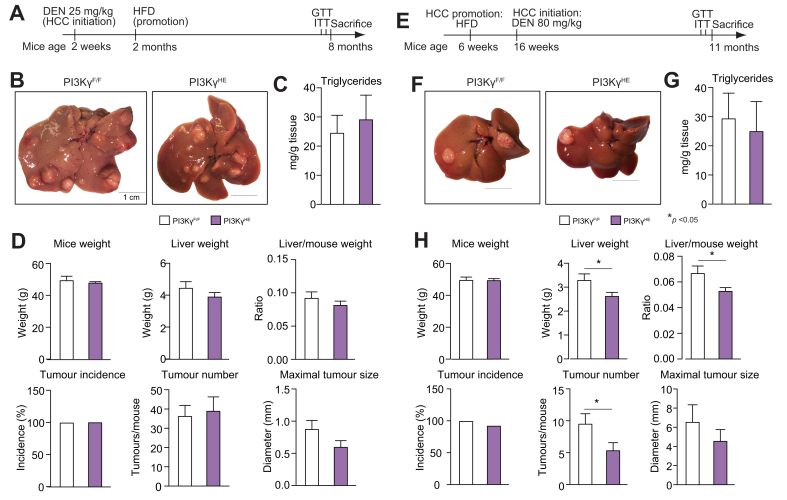
Loss of PI3Kγ in the haematopoietic/endothelial compartments protects mice from obesity-mediated promotion of HCC initiation but not growth. (A) Experimental time-course for the model of obesity promotion of the growth of DEN-initiated HCC. (B) Representative images of livers from 8-month-old PI3Kγ^F/F^ and PI3Kγ^HE^ mice treated as described in (A). (C) Hepatic triglyceride content from the mice in (A). (D) Mice weight, liver weight, liver weight/mouse weight ratio, tumour incidence, tumour number, and maximal tumour size of the PI3Kγ^F/F^ and PI3Kγ^HE^ mice in (A,B). (E) Experimental time-course for the obesity promotion of DEN-driven HCC initiation in PI3Kγ^F/F^ and PI3Kγ^HE^ mice kept on a HFD. (F) Representative images of livers from 11-month-old PI3Kγ^F/F^ and PI3Kγ^HE^ mice treated as described in (E). (G) Hepatic triglyceride content from the mice in (E). (H) Mice weight, liver weight, liver weight/mouse weight ratio, tumour incidence, tumour number, and maximal tumour size of the PI3Kγ^F/F^ and PI3Kγ^HE^ mice described in (E,F). n = 10 mice per group for PI3Kγ^F/F^ and PI3Kγ^HE^ mice in the model described in (A). n = 12–13 mice per group for PI3Kγ^F/F^ and PI3Kγ^HE^ mice in the model described in (E). n = 6 for (C,G). Mann-Whitney test in (C, G, D, H). ∗*p* <0.05. Data are expressed as means, and error bars indicate standard errors. DEN, diethylnitrosamine; HCC, hepatocellular carcinoma; HFD, high-fat diet; PI3Kγ, phosphatidylinositides-3 kinase-gamma.

PI3Kγ^HE^ mice, which lack PI3Kγ specifically in haematopoietic and endothelial cells, largely dissociate PI3Kγ action in leucocytes and endothelial cells from its action in obesity.^13^ Therefore, we have investigated PI3Kγ^HE^ mice and PI3Kγ^F/F^ control mice (PI3Kγ^F/F^) in the models of obesity promotion of HCC growth (Fig. 5A) and initiation (Fig. 5E). The body weight, insulin, and glucose tolerance of PI3Kγ^HE^ mice were similar to PI3Kγ^F/F^ mice (Fig. S7A–H). In contrast to what was observed in PI3Kγ^-/-^ mice (Fig. 1A–G), tumour number and maximal tumour size were not significantly reduced in PI3Kγ^HE^ mice in the model of obesity promotion of HCC growth (Fig. 5A–D). However, similarly to what we observed in *ob/ob*-*PI3K*γ^-/-^ mice (Fig. 1H–J), PI3Kγ^HE^ mice showed a reduced number of carcinomas compared with PI3Kγ^F/F^ mice in the model of obesity-promotion of HCC initiation (Fig. 5E–H).

Overall, PI3Kγ ablation in the haematopoietic and endothelial compartment partially protected obese mice from carcinogen-induced tumour initiation but not from the effects of obesity on the growth of pre-existent HCC. This action of PI3Kγ activity in haematopoietic and endothelial cells on HCC initiation was not associated with differences in lipid content or insulin sensitivity.

### Kupffer cells express a low amount of PI3Kγ protein

**Fig. 6:**
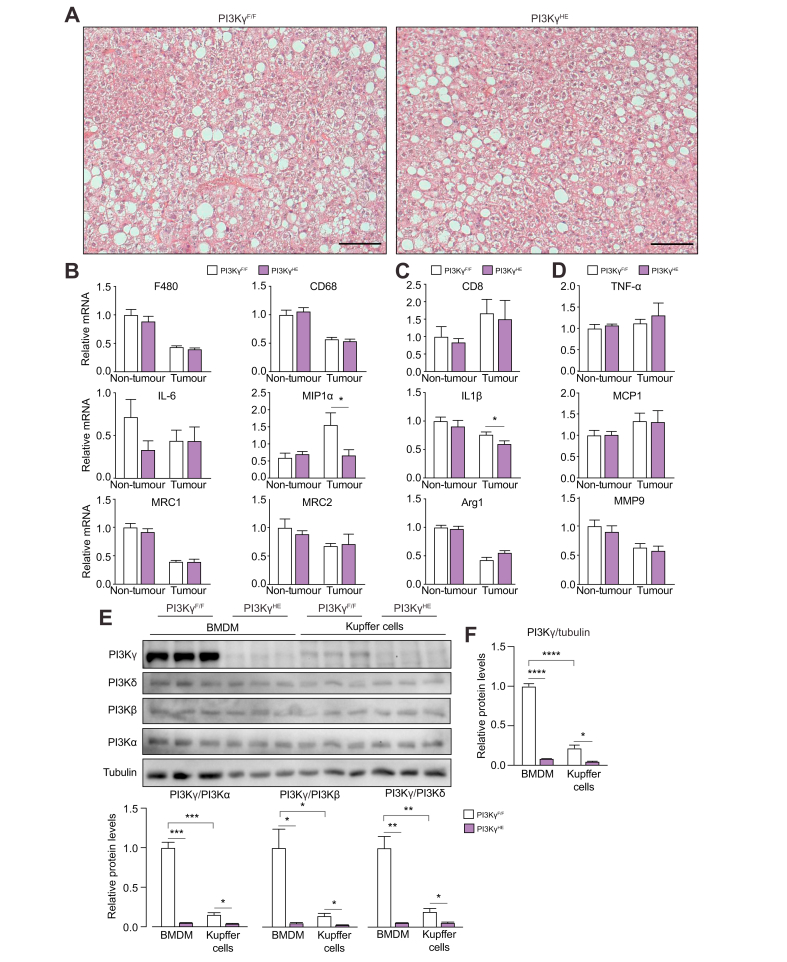
PI3Kγ in haematopoietic/endothelial cells is not required for pro-inflammatory gene expression in the obese liver and HCC. (A) Haematoxylin and eosin staining of liver sections from PI3Kγ^F/F^ and PI3Kγ^HE^ mice are described in Fig. 5A–C (scale bar = 100 μm). (B) mRNA abundance of general macrophage markers in livers from PI3Kγ^F/F^ and PI3Kγ^HE^ mice above. (C) CD8 mRNA abundance in the liver from PI3Kγ^F/F^ and PI3Kγ^HE^ mice above. (D) mRNA abundance of markers of classical or alternative macrophage activation in livers from PI3Kγ^F/F^ and PI3Kγ^HE^ mice above. (E) Immunoblot analysis of protein extracts prepared from BMDM and Kupffer cells of 3-month-old PI3Kγ^F/F^ and PI3Kγ^HE^ mice. (F) Quantifications of the immunoblots in (E). n = 6 mice per group in (A), n = 10 mice per group in (B), n = 3 mice per group in (E,F). Mann-Whitney test in (B, C, D, F). ∗*p* <0.05, ∗∗*p* <0.01, ∗∗∗*p* <0.001, ∗∗∗∗*p* <0.0001. Data are expressed as means, and error bars indicate standard errors. BMDM, bone marrow-derived macrophages; HCC, hepatocellular carcinoma; PI3Kγ, phosphatidylinositides-3 kinase-gamma.

We have analysed steatohepatitis in PI3Kγ^HE^ mice and PI3Kγ^F/F^ mice from the model of obesity promotion of HCC growth (Fig. 5A). Livers from PI3Kγ^HE^ mice developed steatosis to a similar degree to PI3Kγ^F/F^ control animals (Fig. 6A). Furthermore, except for MIP1α and IL-1β expression in carcinoma tissues, PI3Kγ^HE^ mice showed similar mRNA levels of markers of inflammation in normal liver and tumours (Fig. 6B–D). The fact that PI3Kγ ablation in haematopoietic cells caused no substantial effects on inflammatory gene expression in tumours is in apparent contrast with the idea that PI3Kγ ablation in tumour-associated macrophages promotes inflammatory gene expression.[23], [24], [25] Therefore, we have investigated by immunoblot if PI3Kγ was efficiently ablated in Kupffer cells of PI3Kγ^HE^ mice and, as a control, we have analysed bone marrow-derived macrophages (BMDM) from PI3Kγ^HE^ mice and PI3Kγ^F/F^ mice. The results show that PI3Kγ was efficiently deleted in Kupffer cells and BMDM, but we have also found that PI3Kγ protein was far less abundant in Kupffer cells than in BMDM (Fig. 6E,F). Immunoblot analysis of class-1 PI3Ks protein abundance in non-tumour liver and HCC of the mice kept on a HFD from Fig. 5A shows no significant difference in the expression of PI3Kα, PI3Kβ; PI3Kδ, and PI3Kγ between these tissues (Fig. S8A,B). PI3Kγ protein abundance was reduced by more than 70% in non-tumour liver and liver tumours of PI3Kγ^HE^ mice compared with PI3Kγ^F/F^ mice (Fig. S8A,B). Furthermore, the expression of PI3Kγ in liver macrophages was not altered by HFD feeding (Fig. S8C,D). Finally, PI3Kγ protein was efficiently ablated in liver endothelial cells (Fig. S8E).

Overall, our results indicate that PI3Kγ activity in the haematopoietic and endothelial compartment is largely dispensable for inflammatory gene expression in HCC. This observation was explained by our finding that the Kupffer cell displays a low abundance of PI3Kγ.

### Obese PI3Kγ^HE^ mice display reduced DEN-induced liver neutrophil infiltration and hepatocyte proliferation

**Fig. 7:**
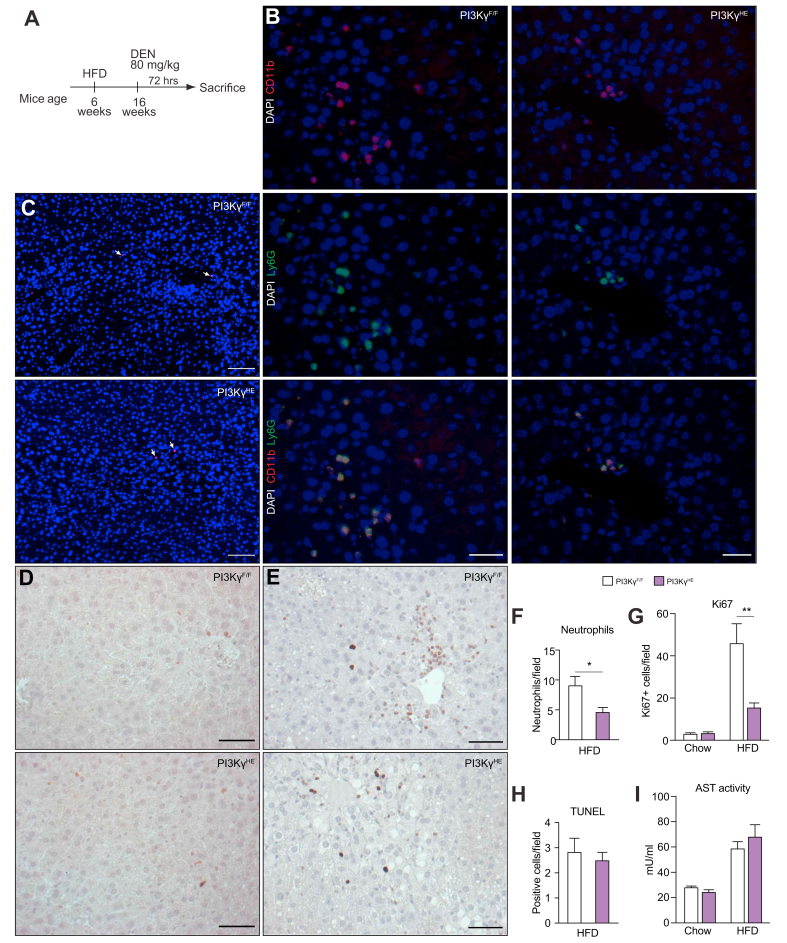
PI3Kγ in haematopoietic/endothelial cells is required for efficient neutrophil recruitment and hepatocyte proliferation induced by DEN administration. (A) Experimental time-course for the acute model of obesity promotion of DEN-driven HCC initiation in PI3Kγ^F/F^ and PI3Kγ^HE^ mice kept on a HFD. (B) Immunostaining of neutrophils in liver sections from 4-month-old PI3Kγ^F/F^ and PI3Kγ^HE^ mice described in (A). Neutrophils are defined as CD11b and Ly6G double-positive cells (scale bar = 50 μm). (C) TUNEL assay of paraffin-embedded sections of livers from PI3Kγ^F/F^ and PI3Kγ^HE^ mice as described in (A) (scale bar = 50 μm). (D) Ki67 staining of liver sections from lean control PI3Kγ^F/F^ and PI3Kγ^HE^ mice 72 h after DEN initiation (scale bar = 50 μm). (E) Ki67 staining of liver sections from obese PI3Kγ^F/F^ and PI3Kγ^HE^ mice 72 h after DEN initiation (scale bar = 50 μm). (F) Quantification of neutrophils from sections in (B). (G) Quantification of the Ki67-positive cells in livers from (D) and (E). (H) Quantification of the number of TUNEL positive cells in (C). (I) AST protein levels in serum from the PI3Kγ^F/F^ and PI3Kγ^HE^ mice 72 h after DEN initiation and their lean controls. n = 7–8 mice per group in (B,F), n = 6 mice per group in (C–E) and (G,H), and n = 6–7 in (I). Mann-Whitney test in (F-I). ∗*p* <0.05, ∗∗*p* <0.01. Data are expressed as means, and error bars indicate standard errors. AST, aspartate aminotransferase; DEN, diethylnitrosamine; HCC, hepatocellular carcinoma; HFD, high-fat diet; PI3Kγ, phosphatidylinositides-3 kinase-gamma; TUNEL, terminal deoxynucleotidyl transferase dUTP nick-end labelling.

The reduced HCC initiation by DEN in ob/ob mice lacking PI3Kγ (Fig. 1H,J) could be explained by reduced neutrophil recruitment to their livers (Fig. 4), which was shown to enhance DEN-induced hepatocyte proliferation promoting HCC initiation.^20^ Hence, we reasoned that the reduced HCC initiation by DEN in obese PI3Kγ^HE^ mice (Fig. 5E–H) could also be associated with reduced neutrophil recruitment. To test this hypothesis, we have investigated the acute induction of neutrophil recruitment and hepatocyte proliferation in PI3Kγ^HE^ mice and PI3Kγ^F/F^ mice made obese by high-fat feeding 72 h after a DEN injection (Fig. 7A). PI3Kγ^HE^ mice showed similar body weight to PI3Kγ^F/F^ mice (Fig. S9A,B). However, neutrophil infiltration and hepatocyte proliferation induced by DEN-injection were reduced in PI3Kγ^HE^ obese mice compared with PI3Kγ^F/F^ controls (Fig. 7). These effects depended on carcinogen administration as we could not detect neutrophils in livers of obese PI3Kγ^HE^ mice and PI3Kγ^F/F^ mice receiving a saline injection, and these mice showed little hepatocyte proliferation with no differences between genotypes (Fig. 7 and Fig. S9C,D). PI3Kγ^HE^ mice and PI3Kγ^F/F^ mice showed similar mRNA abundance of tumour-promoting cytokines after a DEN injection (Fig. S9E). Furthermore, we could not detect neutrophils in livers of lean mice following DEN injection, which showed a marginal hepatocyte proliferation that was not reduced in PI3Kγ^HE^ mice (Fig. 7D,E,G). Compared with chow-fed mice, mice kept on a HFD showed steatosis and increased liver damage following DEN administration (Fig. S10 and Fig. 7C,H,I). However, liver damage, assessed by TUNEL staining of liver sections (Fig. 7C,H, and Fig. S10E,F) and by circulating AST levels (Fig. 7I), was similar between PI3Kγ^HE^ mice and PI3Kγ^F/F^ mice.

These results indicate that PI3Kγ activity in the haematopoietic-endothelial compartment is required for efficient liver infiltration by neutrophils and hepatocyte proliferation acutely induced by DEN administration in obese mice. This phenotype was not associated with differences in body weight, steatosis, or liver damage.

## Discussion

Class-1 PI3Ks are critical targets in cancer therapy, but compound inhibition of all PI3K isoforms causes liver damage, hyperglycaemia, and hyperinsulinaemia as a result of the central role of PI3K in insulin signalling.^9^^,^^11^

We now show that loss of PI3Kγ activity does not affect HCC development in lean mice injected with DEN but reduces the number and the size of tumours in obese mice exposed to DEN.

In the DEN-initiation and HFD-promotion model, PI3Kγ ablation reduced HCC proliferation and growth, in association with reduced liver steatosis and expression of inflammatory cytokines, and reduced insulinaemia. We have reported that the beneficial effects of PI3Kγ inactivation in mice models of diet-induced obesity are mostly the indirect consequence of the reduced adiposity.^13^^,^^14^ Therefore, it is likely that the reduced HCC burden we found in DEN-injected obese mice lacking PI3Kγ is also in part the indirect consequence of their reduced adiposity owing to increased energy expenditure. This idea is consistent with data showing that physical activity reduces HCC proliferation in a DEN-initiation and obesity-promotion mouse model of HCC by reducing insulinaemia, steatosis, and pro-inflammatory signalling.^26^

Although we cannot exclude a role for PI3Kγ activity in the hepatocyte in HCC progression, a major cell-autonomous role for PI3Kγ in HCC progression is not consistent with the fact that PI3Kγ is virtually undetectable in the hepatocyte^12^ and HCC cells,^27^ and that loss of PI3Kγ did not reduce AKT phosphorylation in non-tumour liver and HCC of DEN-injected mice (Fig. 2F,G). Also, the fact that the beneficial effect of PI3Kγ ablation on obesity, insulin resistance, and liver steatosis required an intact leptin signalling,^13^^,^^18^ suggests a possible role for PI3Kγ activity along the adipocyte–brain axis.

Although PI3K ablation did not reduce HCC growth in mice lacking leptin (ob/ob), it significantly reduced HCC number by a mechanism independent of the metabolic effects of PI3K deletion. The reduced number of liver tumours in *ob/ob*-*PI3K*γ^-/-^ mice may be explained by the fact that these mice showed reduced hepatocyte proliferation in association with reduced neutrophil infiltration to their fatty liver acutely induced by DEN. Indeed, a study reported that in mice pups injected with DEN, neutrophils infiltrate into the liver and promote hepatocyte proliferation and HCC by exacerbating oxidative damage.^20^ Consistently with this study, we observed reduced HCC number, hepatocyte proliferation, and liver neutrophil infiltration acutely induced by DEN in adult PI3Kγ^HE^ obese mice. However, in our model, DEN-induced liver neutrophil infiltration was not associated with increased hepatocyte apoptosis. Therefore, PI3Kγ activity in the leucocyte-endothelial compartment promotes HCC initiation by DEN in adult obese mice, possibly, by stimulating hepatocyte proliferation.

We have found that PI3Kγ activity in Kupffer cells is dispensable for the development of steatohepatitis and the expression of inflammatory cytokines in liver tumours of obese mice. Indeed, DEN-injected PI3Kγ^HE^ mice kept on a HFD did not show a gene expression signature indicating increased inflammation within their liver tumours. This observation is in apparent contrast with studies showing that blockage of PI3Kγ activity in macrophages drives a tumour inflammation promoting anti-tumour immunity.[23], [24], [25] However, this apparent discrepancy is explained by our finding that, differently from other macrophages, PI3Kγ does not represent the bulk of PI3K activity in Kupffer cells, and therefore PI3Kγ could be dispensable for the action of PI3K signalling on Kupffer cell activation.

Some limitations of our study should be considered. Our mouse knockout strategy does not distinguish between PI3Kγ activity in different types of leucocytes and endothelial cells. Although PI3Kγ activity in Kupffer cells does not appear to play a significant role in HCC initiation and progression, we cannot exclude a role for PI3Kγ activity in leucocytes other than neutrophils acutely infiltrating the liver in this process (*e.g.* monocytes and lymphocytes). Another limitation is that only male mice have been investigated. This was because female mice are resistant to DEN-induced HCC and diet-induced obesity. Finally, in our model, HCC develops in the absence of cirrhosis. However, obesity promotes HCC independently from cirrhosis.^28^^,^^29^ The value of the model used in our study is of exhibiting increased DEN-induced HCC initiation and progression in association with obesity, steatosis, metabolic inflammation, and hyperinsulinaemia.

Overall, our results indicate that PI3Kγ activity in the leucocyte and endothelial cell-compartment is required for efficient acute induction of neutrophil liver infiltration, hepatocyte compensatory proliferation, and HCC initiation induced by DEN in obese mice; whereas PI3Kγ activity outside the haematopoietic-endothelial compartment drives metabolic mechanisms promoting weight-gain, hyperinsulinaemia, steatosis, and inflammatory gene expression by which obesity promotes HCC growth.

It is concluded that PI3Kγ-selective inhibition may reduce HCC initiation and slow HCC progression in obese subjects. Future clinical studies will test this possibility.

## Financial support

10.13039/501100002794Cancerfonden (CAN2017/472 and 20 0840 PjF); 10.13039/501100001648EFSD, Diabetes and Cancer Research Programme; a Swiss National Science Foundation, Sinergia grant 154499, and project grant 152998; the 10.13039/501100004359Swedish Research Council grant 2014-3019; the 10.13039/501100009708Novo Nordisk Fonden (NNF19OC0057174); and the 10.13039/501100008550Diabetesfonden (DIA 2018-384 and DIA2020-564) to G.S.

## Authors’ contributions

Performed most of the experiments: BB. Performed experiments: LB, CS. Performed some experiments: FZ. Backcrossed PI3Kγ mice into the ob/ob background: FZ. Analysed data: BB, LB, CS. Contributed to the manuscript writing: BB. Contributed to the preparation of figures: LB, CS. Conceived the project, directed the study, contributed to data interpretation, and wrote the manuscript: GS. Read and approved the manuscript: all authors.

## Data availability statement

Datasets will be made available by the corresponding author upon reasonable request.

## Conflicts of interest

The authors declare they have no competing interests to disclose.

Please refer to the accompanying ICMJE disclosure forms for further details.
