# Supplementary material for: PI3Kγ promotes obesity-associated hepatocellular carcinoma by regulating metabolism and inflammation
Source: JHEP Rep. 2021 Sep 2;3(6):100359. doi: 10.1016/j.jhepr.2021.100359 (PMC8521290; doi:10.1016/j.jhepr.2021.100359)
Supplement: Multimedia component 1 [file mmc1.pdf]

PI3Kγ promotes obesity-associated hepatocellular carcinoma by regulating metabolism and inflammation

Barbara Becattini, Ludovic Breasson, Claudia Sardi, Fabio Zani, Giovanni Solinas

Table of contents

Supplementary materials and methods....2

Table S1....5

Fig. S1....6

Fig. S2....7

Fig. S3....8

Fig. S4....10

Fig. S5....11

Fig. S6....13

Fig. S7....14

Fig. S8....16

Fig. S9....17

Fig. S10....19

Supplementary references....21

SUPPLEMENTARY MATERIALS AND METHODS

In vivo studies

Mice used for this study were males C57BL/6J. Mice were maintained at specific pathogen-free conditions at room temperature of 22 ˚C under 12 hours of light and dark cycles. Experiments were authorized by the Veterinary committee of canton Fribourg and the Research Animal Ethics Committee of the University of Gothenburg. PI3K$\gamma^{-/-}$ mice; ob/ob-PI3K$\gamma^{-/-}$ mice, and PI3Kγ^{HE} mice were previously described[1,2]. PI3Kγ^{HE} mice were obtained by crossing PI3K$\gamma^{F/F}$ mice with mice expressing the Cre recombinase under the control of the Tek promoter, which causes a complete deletion in hematopoietic and endothelial cells[1]. *ob/ob-PI3K*$\gamma^{-/-}$ mice and control *ob/ob* mice have been previously described[1].

For the model of diet-induced obesity promoting the growth of DEN-initiated hepatocellular carcinoma (Fig. 1A and 5A), 25 mg/kg DEN were injected intraperitoneally into 2-week-old mice. At the age of 2 months, mice were separated into two dietary groups and fed either a chow diet or a high-fat diet (Bio-Serv, F3282) until the age of 8 months, when mice were sacrificed. For the model of obesity promotion of DEN-initiation, 16-week-old *ob/ob-PI3K*$\gamma^{-/-}$ mice and control *ob/ob* mice; or 16-week-old PI3Kγ^{HE} mice and PI3K$\gamma^{F/F}$ mice were injected intraperitoneally with DEN and kept on a chow diet until the age of 11 months when the mice were sacrificed (Fig 1H and Fig. 5E). For glucose and insulin tolerance tests, mice were fasted for 4 hours in the morning and were injected with 1 g/kg body weight (glucose tolerance test) and 1 IU insulin /kg (for insulin tolerance test). Blood glucose was measured using a commercial glucometer from one drop of blood collected from the tail tip. At sacrifice, the mouse liver was removed and analyzed under a stereo microscope, tumors were counted to evaluate tumor number and the width of the largest tumors was measured.

Histology immunostaining

Liver and adipose tissues were fixed in 4% buffered formaldehyde and embedded in paraffin. Antigen retrieval on tissue sections, previously deparaffinized and rehydrated, was performed with S1700 (Dako) in a pressure cooker. For neutrophil staining, tissue sections were blocked with 5% bovine serum albumin in PBS for 1 hour at room temperature and stained with Ly6G (1:50, 1A8, 551459 BD Bioscience) and CD11b (1:50, ab133357 Abcam) overnight at 4°C in blocking solution. Slides were washed in PBS and incubated for 1 hour at room temperature with Alexa Fluor 488/ 594–conjugated antibody (1:200, Invitrogen A-11006 and Jackson 711-586-152). Positive neutrophils are defined as $CD11b^{+}Ly6G^{+}$ cells. Cell nuclei were stained with DAPI, and neutrophils were counted in three random fields per section. For Mac2, F4-80, and Ki67 staining endogenous peroxidase was blocked with 3% of H_2O_2 for 20 min at room temperature, and nonspecific sites were blocked with horse serum (Vector Laboratories, Burlington, CA, USA) 1:75 in PBS, 20 min at room temperature. Tissue sections were then incubated overnight at high relative humidity at 4°C with anti-MAC2 (Cederlane), F4-80 (Biorad), or Ki67 (Cell Signaling) at a final dilution of 1:2000 (MAC2) or 1:500 in PBS, washed with PBS and incubated with IgG biotinylated anti-mouse -rat and -rabbit secondary antibody respectively (Vector Laboratories) diluted 1:200 in PBS, for 30 min at room temperature. Tissue sections were then washed in PBS and incubated in ABC reagent (Vector Laboratories) for 60 min at room temperature, washed in PBS, and incubated with Sigma Fast 3,3'-diaminobenzidine as substrate; counterstained with hematoxylin, dehydrated, and mounted with coverslips. Images were acquired by optical microscopy, and positive cells were counted in three random fields per section. TUNEL assay was performed accordingly to manufacturer's protocol (TMR Red, Roche).

Molecular analysis

RNA was extracted using Guanidinium thiocyanate-phenol-chloroform protocol retrotranscribed and mRNA abundances of specific genes were measured by real-time PCR using cyclophilin as housekeeping gene (Supplementary information table S1), and SYBR green

mix (Biorad) as previously described [1]. For immunoblotting, frozen liver tissues were pulverized in liquid N_2 using a mortar and pestle. An aliquot was weighed and lysed in lysis buffer (20mM Tris-HCl, 5% glycerol, 138 mM NaCl, 2.7 mM KCl, 1% NP-40, 5 mM EDTA) with phosphatases and proteases inhibitors. Protein concentrations were determined using Protein Assay Dye (Pierce, ThermoScientific) and BSA as standard. Protein extracts were denatured in SDS sample buffer, resolved by SDS-PAGE, and transferred onto polyvinylidene fluoride (PVDF) transfer membrane (GE Healthcare) in Tris Base/Glycine buffer. The membrane was blocked with 3% BSA, 0.3% Tween-20, in PBS and then probed with the indicated antibody. Primary antibodies were from Cell Signaling except PI3Kγ (Russian Wymann Lab) and PI3Kδ (Millipore). Secondary antibodies were from GE healthcare. Antibodies binding to the blotting membrane were visualized using the Immobilon Western Chemiluminescence HRP substrate (Millipore) and ChemiDoc Touch Imaging System (Bio-Rad). Signal intensities were quantified by densitometric analyses using Image Lab software (version 5.2.1; Bio-Rad). Serum insulin was measured by Elisa according to the manufacturer's instructions (Crystal Chem #90080). Liver triglycerides and serum aspartate aminotransferase (AST) levels were measured by commercial assays (Abcam: ab65336 and ab105135, respectively).

Statistical methods

Data are expressed as means, and error bars indicate standard errors. P values were calculated by either Wilcoxon–Mann-Whitney test or Student's T-test for simple comparison and by two-way ANOVA followed by Sidak when two different categorical variables are considered (such as for the glucose tolerance test and insulin tolerance test curves). $P<0.05$ is considered statistically significant. Statistical analysis was performed with GraphPad Prism.

Table S1. Supplemental Methods: List of Primers used for real-time quantitative PCR.

Name	Forward	Reverse
Cyclophilin (housekeeping gene)	ATG GTC AAC CCC ACC GTG T	TTT CTG CTG TCT TTG GAA CTT TGT C
F4/80	CTT TGG CTA TGG GCT TCC AGT C	GCA AGG AGG ACA GAG TTT ATC GTG
CD68 (Macrosialin)	CCT CGC CTA GTC CAA GGT C	GGA TTC GGA TTT GAA TTT GGG CT
CD11c (Itgax)	CTG GAT AGC CTT TCT TCT GCT G	GCA CAC TGT GTC CGA ACT C
IL-1β	GCA ACT GTT CCT GAA CTC AAC T	TCT TTT GGG GTC CGT CAA CT
TNF-α	CCC CAA AGG GAT GAG AAG TT	CTC CTC CAC TTG GTG GTT TG
MIP-1α (CCL3)	TTC TCT GTA CCA TGA CAC TCT GC	CGT GGA ATC TTC CGG CTG TAG
MCP-1 (CCL2)	CCC CAA GAA GGA ATG GGT CC	GGT TGT GGA AAA GGT AGT GG
IL-6	TCC TAC CCC AAT TTC CAA TGC TC	TTG GAT GGT CTT GGT CCT TAG CC
IL-1Ra	AAA TCT GCT GGG GAC CCT AC	TCT TCT AGT TTG ATA TTT GGT CCT TG
CCR2	GCC ATA CCT GTA AAT GCC ATG C	GGC AGG ATC CAA GCT CCA AT
RANTES	GCT GCT TTG CCT ACC TCT CC	TCG AGT GAC AAA CAC GAC TGC
MMP-9	CAT TCG CGT GGA TAA GGA GT	CAC TGC AGG AGG TCG TAG G
MGL-1	TGA GAA AGG CTT TAA GAA CTG GG	GAC CAC CTG TAG TGA TGT GGG
Arg-1	CTC CAA GCC AAA GTC CTT AGA G	AGG AGC TGT CAT TAG GGA CATC
MRC-2	TAC AGC TCC ACG CTA TGG ATT	CAC TCT CCC AGT TGA GGT ACT
MRC-1	TGA TTA CGA GCA GTG GAA GC	GTT CAC CGT AAG CCC AAT TT
CD8	AAG AAA ATG GAC GCC GAA CTT	AAG CCA TAT AGA CAA CGA AGG TG
iNOS	CAG CTG GGC TGT ACA AAC CTT	CAT TGG AAG TGA AGC GTT TCG
Cyclin D1 (CCND1)	GGG TGG GTT GGA AAT GAA C	TCC TCT CCA AAA TGC CAG AG

Fig. S1

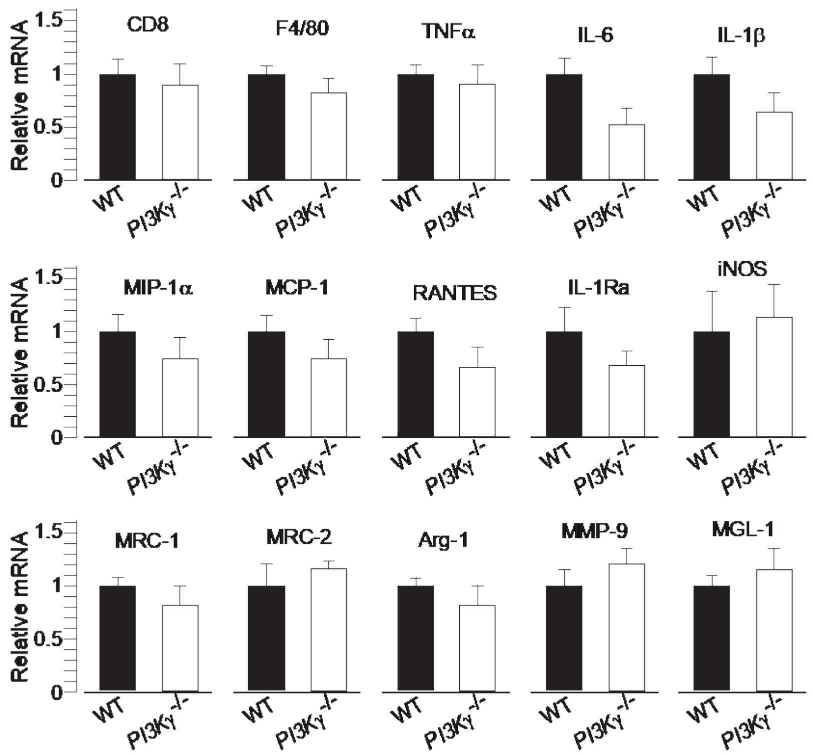

Fig. S1. **PI3Kγ ablation does not affect the mRNA abundance of leukocyte markers in HCCs from lean mice.**

Real-time qPCR analysis of the mRNA abundance of leukocyte markers (CD8 and F4/80) and of markers of classical "M1" or alternative "M2" macrophage activation in HCCs from the lean WT and PI3K$\gamma^{-/-}$ mice kept on chow diet described in figure 1. n = 5-8 mice per group. Data are expressed as mean, and error bars indicate standard errors.

Fig. S2

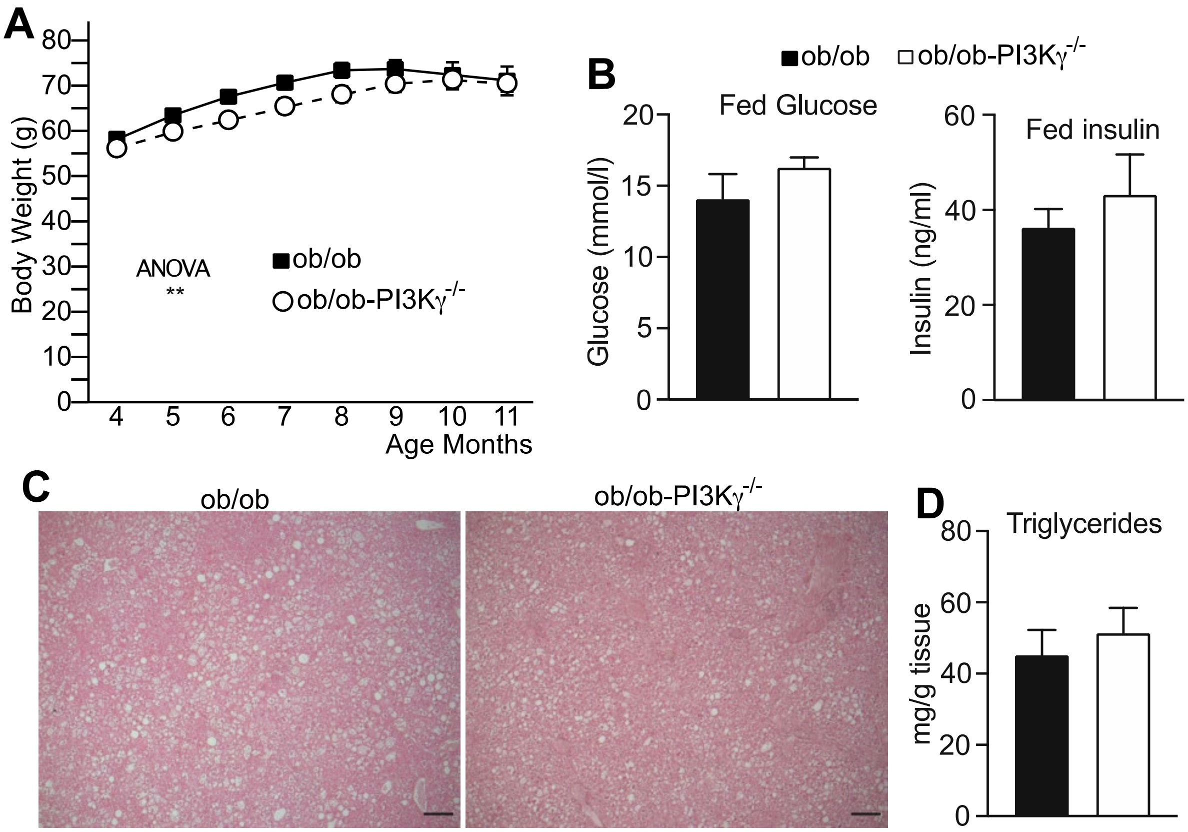

Fig. S2. Metabolic phenotype of ob/ob and ob/ob-PI3K$\gamma^{-/-}$ mice. (A) Growth curves of the mice described in figure 1g. (B) Fed blood glucose and serum insulin levels of 11 months old ob/ob and ob/ob-PI3K$\gamma^{-/-}$ mice in figure 1H. (C) Hematoxylin and eosin staining of liver sections from the 11-month-old ob/ob and ob/ob-PI3K$\gamma^{-/-}$ mice described in figure 1H (Scale bar = 100 μm). (D) Triglyceride content in livers from the mice described above. N = 14 mice per group in A, n = 4 per group in B, n = 8 per group in C, n = 6 per group in D. **P < 0.01. Data are expressed as mean, and error bars indicate standard errors.

Fig. S3

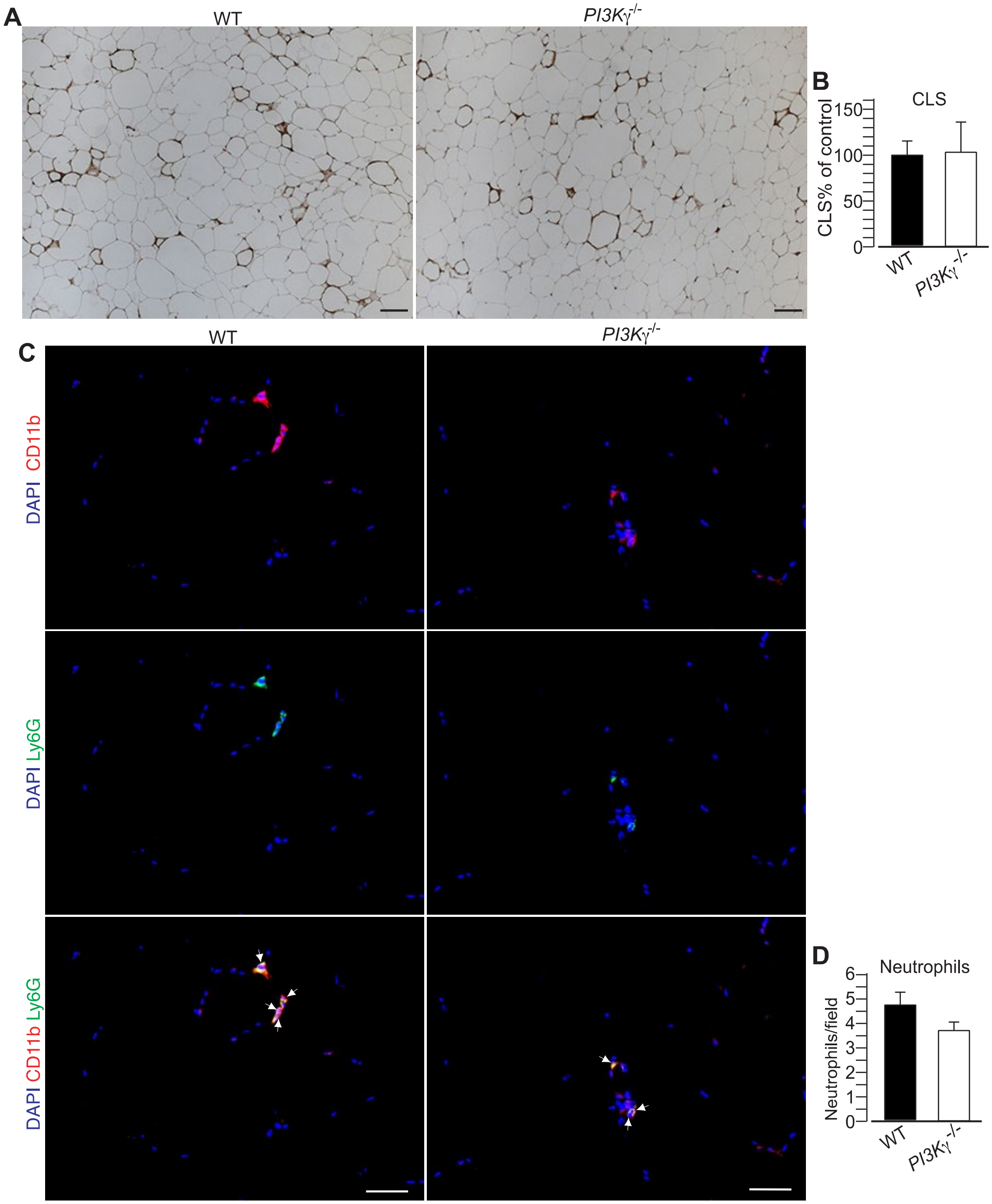

Fig. S3. Effects of PI3Kγ ablation on adipose tissue myeloid cell recruitment in DEN-injected 8-month-old mice. (A) Mac2 immuno-staining of adipose tissue sections

of WT and PI3K$\gamma^{-/-}$ mice kept on high-fat diet described in figure 3 (Scale bar = 100 μm).
(B) Quantification of the number of crown-like structures (CLS) in A.
(C) CD11b and Ly6G co-staining of adipose tissue sections from the WT and PI3K$\gamma^{-/-}$ mice described above. Adipose tissue neutrophils are defined as CD11b and Ly6G double-positive cells (Scale Bar = 100 μm). (D) Quantification of the number of adipose tissue neutrophils from C. n = 7 mice per group in A, n = 9-10 mice per group in C. Data are expressed as mean, and error bars indicate standard errors.

Fig. S4

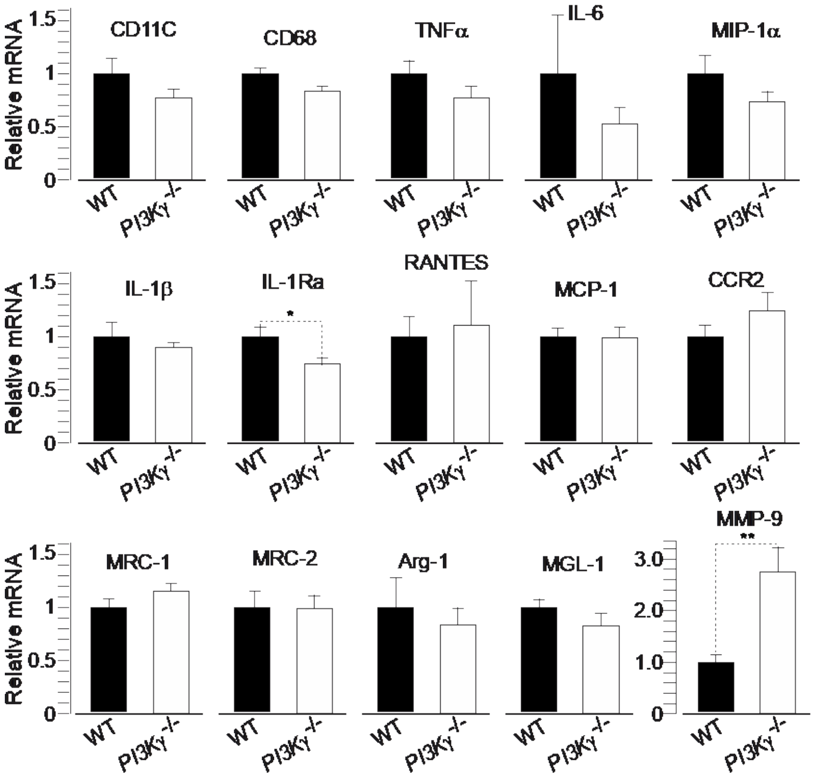

Fig. S4. Effects of PI3Kγ ablation on adipose tissue gene expression in DEN-injected 8-month-old mice. Real-time qPCR analysis of the mRNA abundances of macrophage markers and of markers of classical "M1" or alternative "M2" macrophage activation in adipose tissues from the obese WT and PI3Kγ$^{-/-}$ mice kept on high-fat diet described in figure 3. n = 8-9 mice per group. *P < 0.05, **P < 0.01. Data are expressed as mean, and error bars indicate standard errors.

Fig. S5

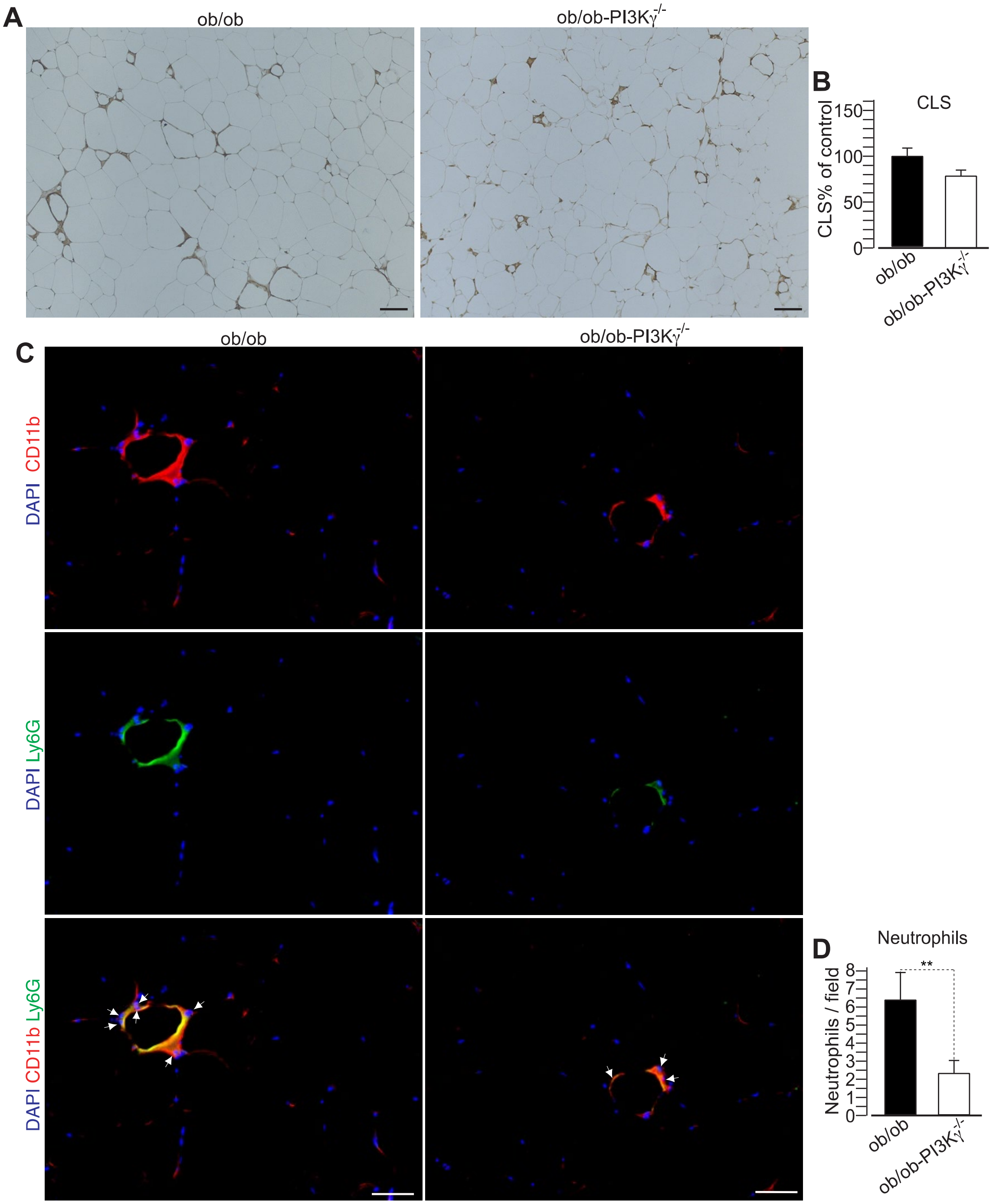

Fig. S5. Effects of PI3Kγ ablation on adipose tissue myeloid cell recruitment in DEN-injected 11-month-old *ob/ob* and *ob/ob*-PI3Kγ$^{-/-}$ mice. (A) Mac2 immuno-staining of adipose tissue sections of the 11-month-old ob/ob and ob/ob-PI3Kγ-/- mice described in figure 1G (Scale bar = 100 μm). (B) Quantification of the number of crown-

like structures (CLS) in A. (C) CD11b and Ly6G co-staining of adipose tissue sections from the ob/ob and ob/ob-PI3Kγ-/- mice described above. Adipose tissue neutrophils are defined as CD11b and Ly6G double-positive cells (Scale bar = 100 μm). (D) Quantification of the number of adipose tissue neutrophils from C. n = 9 mice per group. **P < 0.01. Data are expressed as mean, and error bars indicate standard errors.

Fig. S6

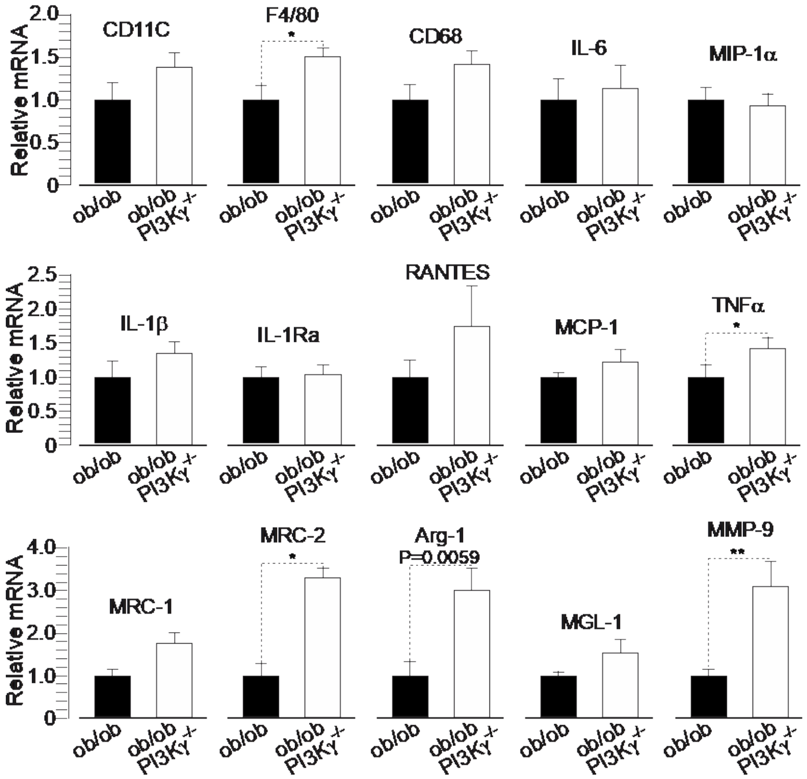

Fig. S6. Effects of PI3Kγ ablation on adipose tissue gene expression in DEN-injected 11-month-old *ob/ob* and *ob/ob*-PI3Kγ$^{-/-}$ mice. Real-time qPCR analysis of the mRNA abundances of macrophage markers and of markers of classical "M1" or alternative "M2" macrophage activation in adipose tissues from the *ob/ob* and *ob/ob*-PI3Kγ$^{-/-}$ mice described in figure S5. n = 6-8 mice per group. *P < 0.05, **P < 0.01. Data are expressed as mean, and error bars indicate standard errors.

Fig. S7

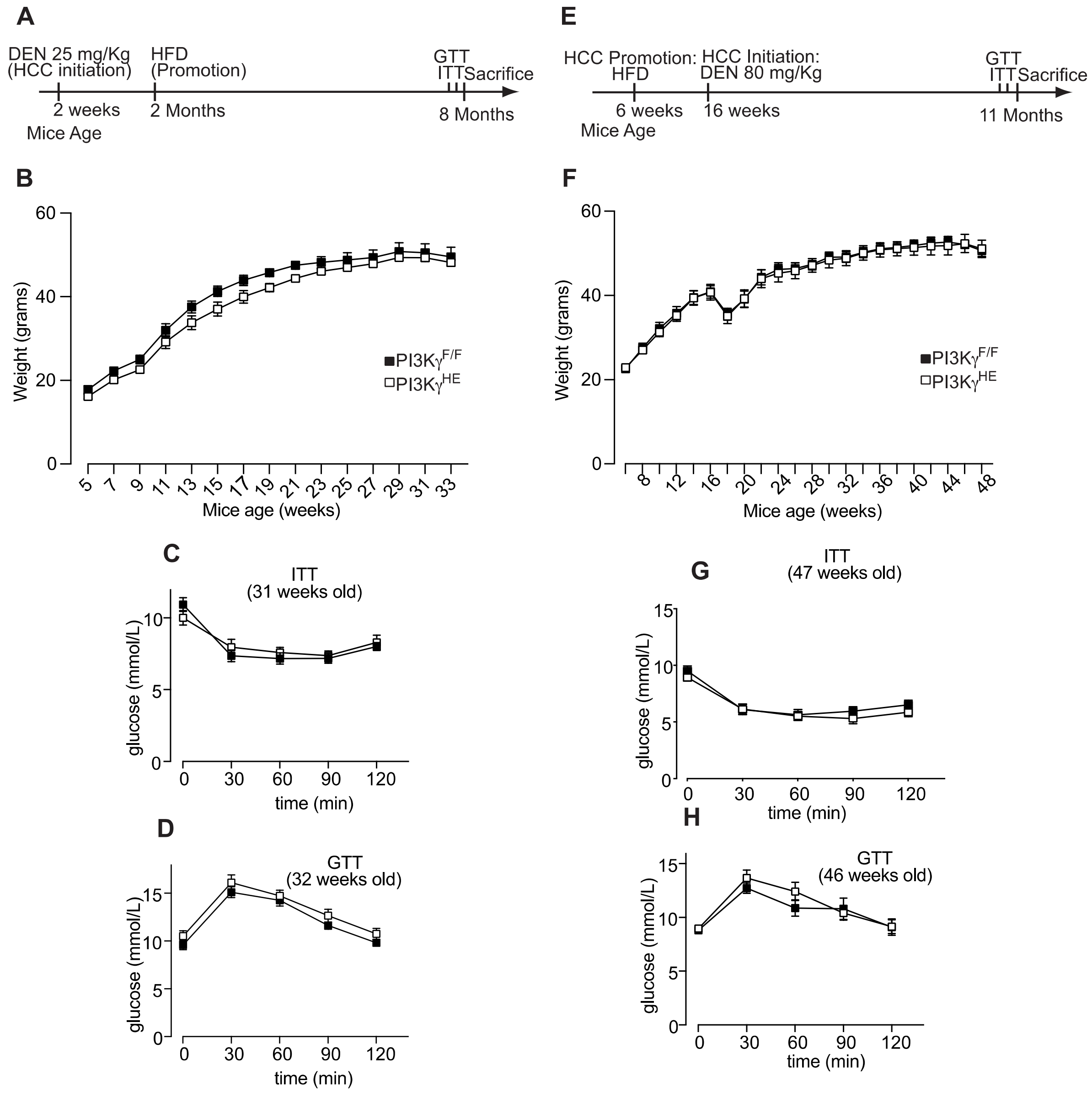

Fig. S7. Loss of PI3Kγ in hematopoietic and endothelial cells does not affect body weight or insulin sensitivity in models of obesity-promoted HCC. (A) Experimental time-course for the model of diet-induced obesity promoted the growth of DEN-initiated hepatocellular carcinoma (HCC). (B) Growth curves of PI3Kγ$^{F/F}$ and PI3Kγ^{HE} mice placed on high-fat diet as described in A. (C) Insulin tolerance test of the mice in B at the age of 31 weeks. (D) Glucose tolerance test of the mice in B at the age of 32 weeks. (E) Experimental time-course for the obesity promotion of DEN-driven HCC initiation in PI3Kγ$^{F/F}$ and PI3Kγ^{HE} mice kept on HFD. (F) Growth curves of PI3Kγ$^{F/F}$ and PI3Kγ^{HE}

mice placed on high-fat diet as described in E. (G) Insulin tolerance test of the mice in F at the age of 47 weeks. (H) Glucose tolerance test of the mice in F at the age of 46 weeks. n = 10 mice per group in B, n = 8 mice per group in C-D, n = 12-13 mice per group in F, n = 8-12 mice per group in G-H. Data are expressed as mean, and error bars indicate standard errors.

Fig. S8

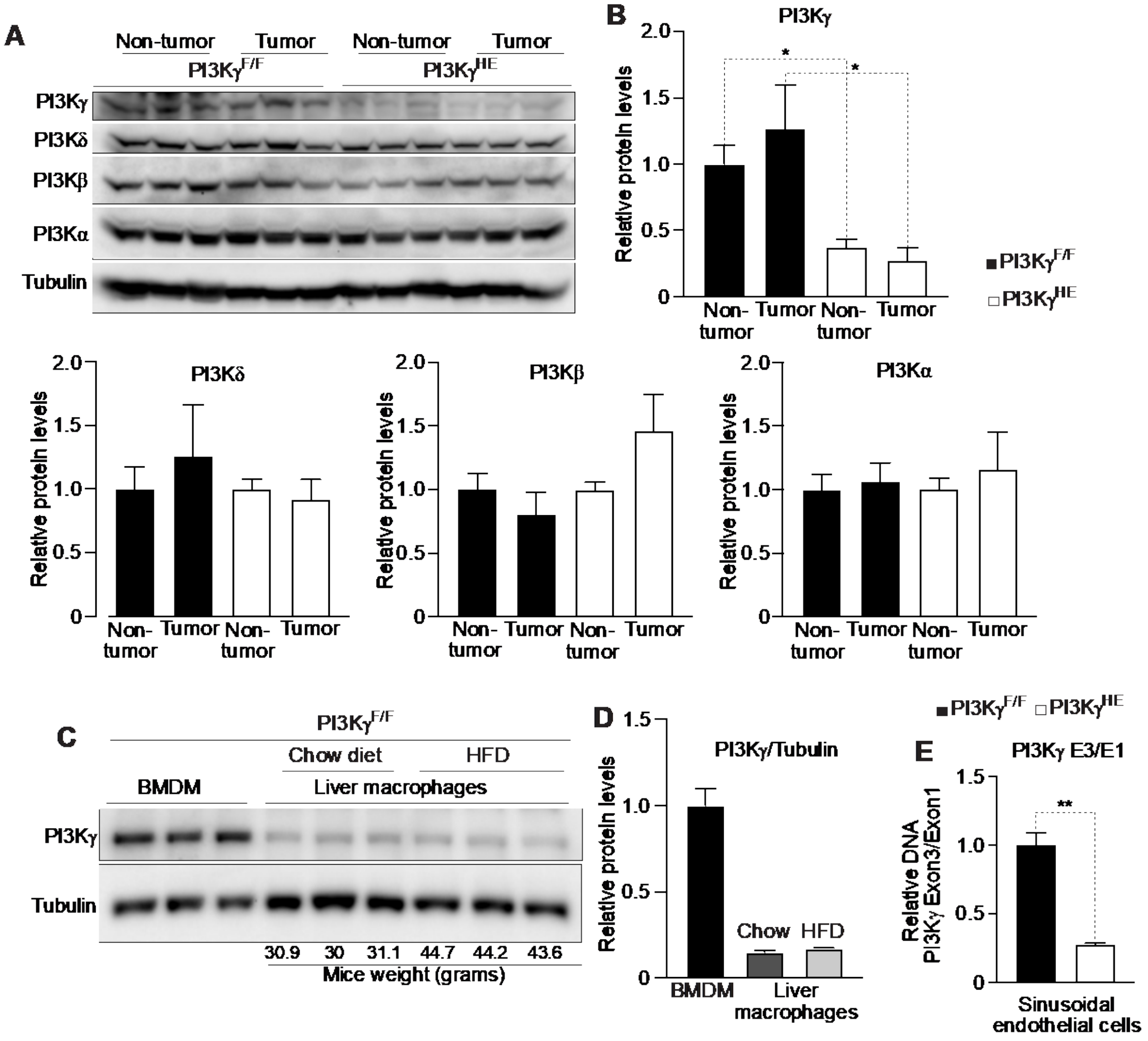

Fig. S8. Hepatic expression of class-1 PI3Ks.

(A) Immunoblot analysis of protein extracts obtained from non-tumor liver and HCC of 8-month-old PI3Kγ$^{F/F}$ and PI3Kγ^{HE} obese mice. (B) Quantifications of the immunoblots in A. (C) Immunoblot analysis of protein extracts prepared from BMDM and liver macrophages from PI3Kγ$^{F/F}$ mice kept in chow or HFD. (D) Quantifications of the immunoblots in C. (E) Real-time qPCR analysis of PI3Kγ exon-3 deletion as measured by the ratio between exon-3 and exon-1. n = 3 mice per group. *P < 0.05, **P < 0.01. Data are expressed as mean, and error bars indicate standard errors.

Fig. S9

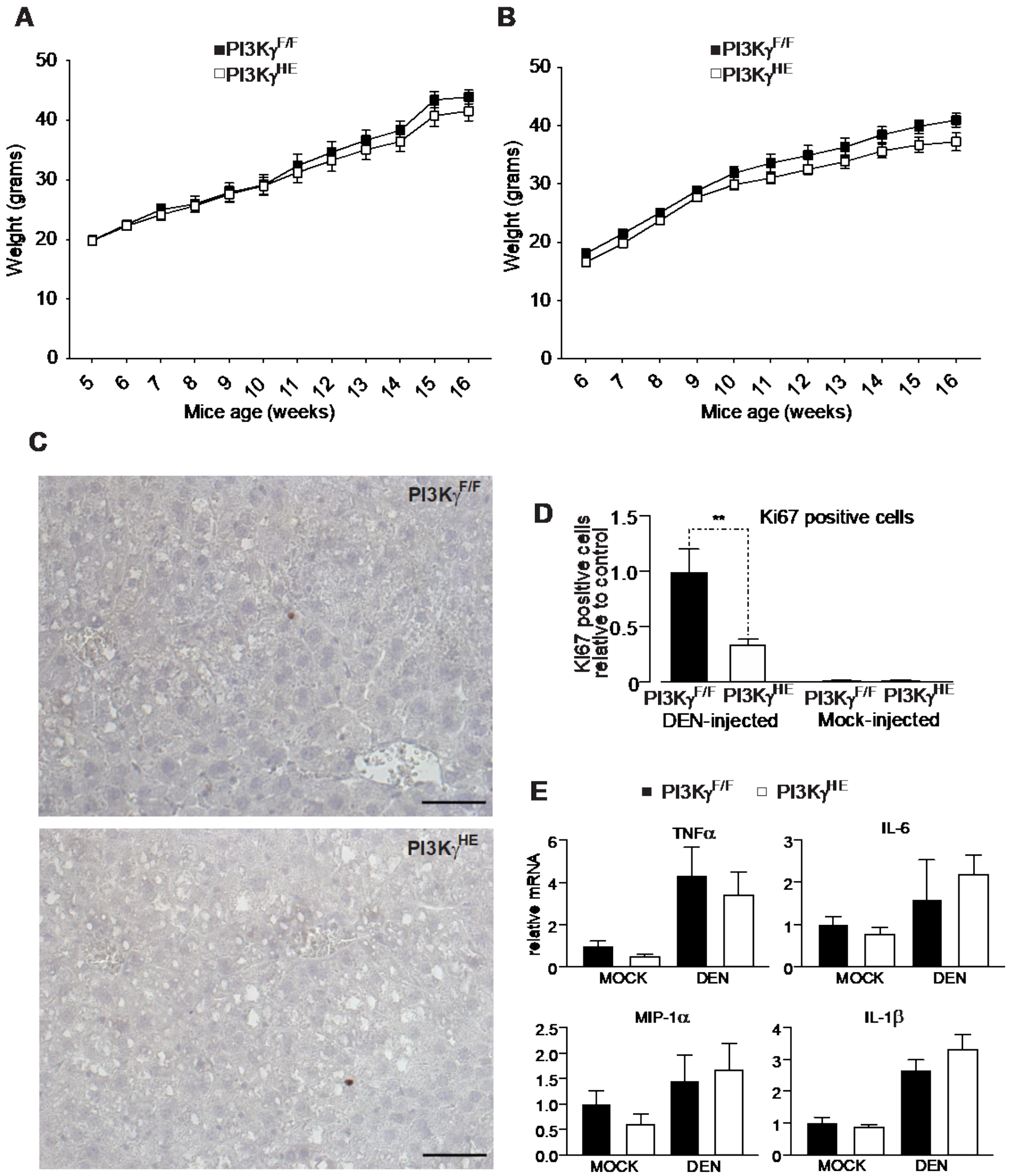

Fig. S9. Growth curves, basal hepatocyte proliferation, and expression of tumor-promoting cytokines of the mice in Fig. 7. (A) Growth curves of PI3Kγ$^{F/F}$ and PI3Kγ^{HE} mice placed on a high-fat diet and sacrificed 72 hours after DEN initiation. (B) Growth curves of PI3Kγ$^{F/F}$ and PI3Kγ^{HE} mice placed on a high-fat diet and sacrificed 72 hours after mock injection. (C) Ki67 staining of liver sections from PI3Kγ$^{F/F}$ and PI3Kγ^{HE} mice described in B 72 hours after mock injection (Scale bar = 50 μm). (D) Quantification of

the Ki67 positive cells in C relative to DEN injected mice (data in Fig. 7E. (E) mRNA abundance of markers of classical macrophage activation in livers from PI3K$\gamma^{F/F}$ and PI3Kγ^{HE} mice 72 hours after DEN (80mg/Kg) or mock injection. n = 7-9 mice per group in A, n = 8 per group in B, n = 7 mice per group in C, n = 6-7 mice per group in D. **P < 0.01. Data are expressed as mean, and error bars indicate standard errors.

Fig. S10

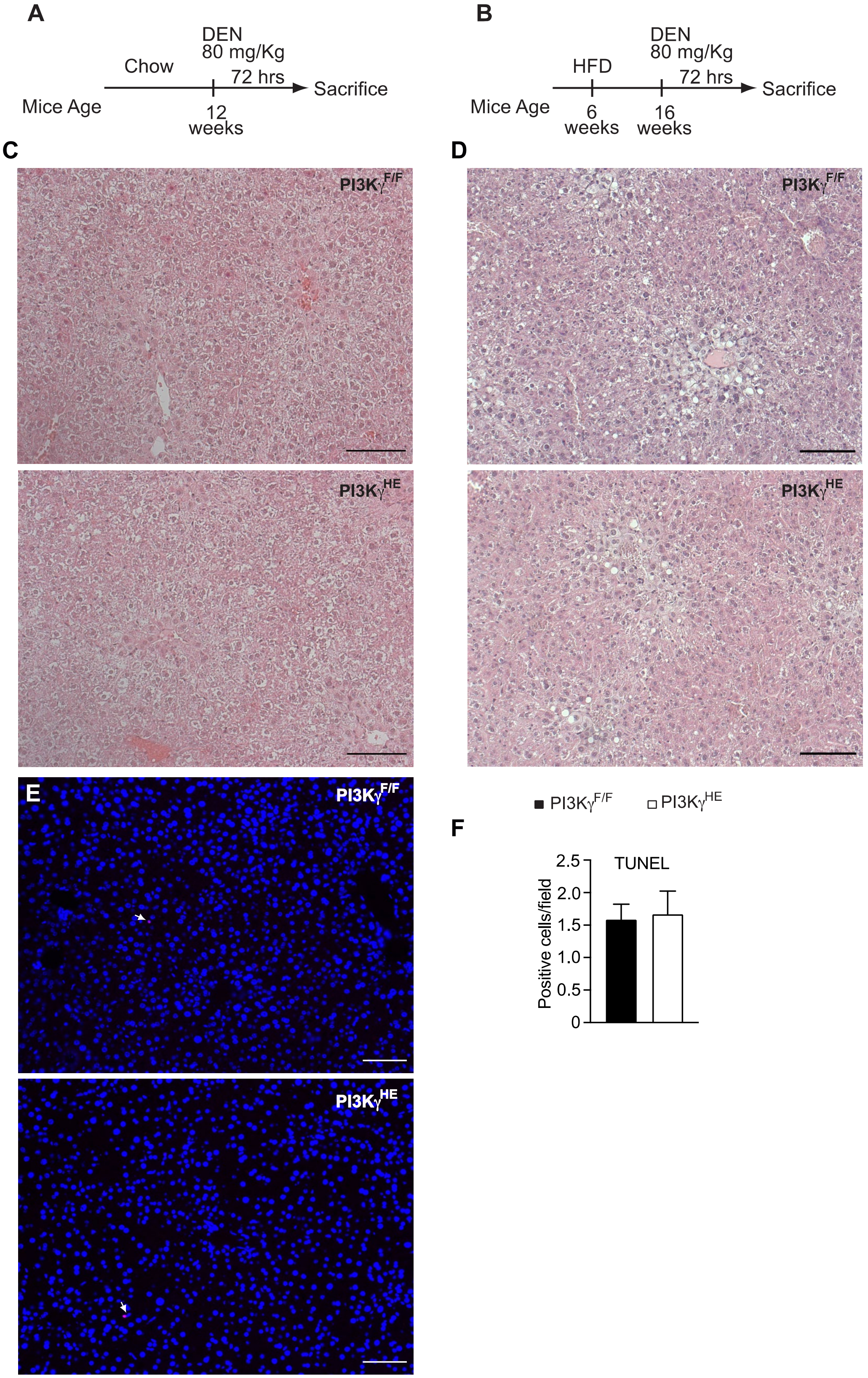
A
DEN
80 mg/Kg
Chow
72 hrs
Sacrifice
Mice Age
12
weeks
B
DEN
80 mg/Kg
HFD
72 hrs
Sacrifice
Mice Age
6
weeks
16
weeks
C
D
PI3Kγ F/F
PI3Kγ HE
E
PI3Kγ F/F
PI3Kγ HE
F
■ PI3Kγ F/F
□ PI3Kγ HE
TUNEL
Positive cells/field
2.5
2.0
1.5
1.0
0.5
0

Fig. S10. Steatosis and apoptosis in livers of PI3K$\gamma^{F/F}$ and PI3Kγ^{HE} mice following an acute DEN injection, (related to figure 7).

(A) Experimental time-course for the acute model of obesity promotion of DEN-driven HCC initiation in PI3K$\gamma^{F/F}$ and PI3Kγ^{HE} mice kept on chow diet. (B) Experimental time-course for the acute model of obesity promotion of DEN-driven HCC initiation in PI3K$\gamma^{F/F}$ and PI3Kγ^{HE} mice kept on HFD. (C) Hematoxylin and eosin staining of liver sections from PI3K$\gamma^{F/F}$ and PI3Kγ^{HE} mice described in A (Scale bar = 100 μm). (D) Hematoxylin and eosin staining of liver sections from PI3K$\gamma^{F/F}$ and PI3Kγ^{HE} mice described in B (Scale bar = 100 μm). (E) TUNEL assay of paraffin-embedded sections of livers from PI3K$\gamma^{F/F}$ and PI3Kγ^{HE} mice described in A (Scale bar = 50 μm). (F) Quantification of the number of TUNEL positive cells in E. Data are expressed as mean, and error bars indicate standard errors.

Supplementary references

[1] Breasson L, Becattini B, Sardi C, Molinaro A, Zani F, Marone R, et al. PI3Kgamma activity in leukocytes promotes adipose tissue inflammation and early-onset insulin resistance during obesity. Sci Signal 2017;10.

[2] Becattini B, Marone R, Zani F, Arsenijevic D, Seydoux J, Montani JP, et al. PI3Kgamma within a nonhematopoietic cell type negatively regulates diet-induced thermogenesis and promotes obesity and insulin resistance. Proc Natl Acad Sci U S A 2011;108:E854-863.
