# Supplementary material for: PI3Kγ promotes obesity-associated hepatocellular carcinoma by regulating metabolism and inflammation
Source: JHEP Rep. 2021 Sep 2;3(6):100359. doi: 10.1016/j.jhepr.2021.100359 (PMC8521290; doi:10.1016/j.jhepr.2021.100359)
Supplement: Multimedia component 2 [file mmc2.pdf]

JOURNAL
OF HEPATOLOGY

Journal of Hepatology
CTAT methods

Tables for a "Complete, Transparent, Accurate and Timely account" (CTAT) are now mandatory for all revised submissions. The aim is to enhance the reproducibility of methods.

- Only include the parts relevant to your study
- Refer to the CTAT in the main text as 'Supplementary CTAT Table'
- Do not add subheadings
- Add as many rows as needed to include all information
- Only include one item per row

If the CTAT form is not relevant to your study, please outline the reasons why:

1.1 Antibodies

Name	Citation	Supplier	Cat no.	Clone no.
P-Erk 1/2		Cell Signaling	4370	
Erk 1/2		Cell Signaling	9102	
P-AKT		Cell Signaling	4060	
AKT		Cell Signaling	9272	
P-p38		Cell Signaling	4511	
p38		Cell Signaling	8690	
PI3Kγ		Wymann Lab	Gift	
PI3Kδ		Millipore	04-401	
PI3Kβ		Cell Signaling	3011	
PI3Kα		Cell Signaling	4249	
Tubulin (α/β)		Cell Signaling	2148	
Ki67		Cell Signaling	12202	
F4/80		Bio-Rad	MCA497R	
MAC-2		Cederlane	CL8942AP	
CD11b		Abcam	ab133357	
Ly-6G		BD biosciences	551459	

1.2 Cell lines

Name	Citation	Supplier	Cat no.	Passage no.	Authentication test method

1.3 Organisms

Name	Citation	Supplier	Strain	Sex	Age	Overall n number
WT mice		Wymann Lab	C57BL/6	Male	8 months old	22
PI3K$\gamma^{-/-}$ mice		Wymann Lab	C57BL/6	Male	8 months old	17
ob/ob mice		Jackson	C57BL/6	Male	4-11 months old	18

ob/ob PI3Kγ $^{-/-}$ mice		Solinas Lab	C57BL/6	Male	4-11 months old	20
PI3Kγ $^{F/F}$ mice		EUCOMM	C57BL/6	Male	4-11 months old	47
PI3Kγ ^{HE} mice		Wymann Lab	C57BL/6	Male	4-11 months old	44

1.4 Sequence based reagents

Name	Sequence forward	Sequence reverse	Supplier
Cyclophilin (housekeeping gene)	ATG GTC AAC CCC ACC GTG T	TTT CTG CTG TCT TTG GAA CTT TGT C	Eurofins genomics
F4/80	CTT TGG CTA TGG GCT TCC AGT C	GCA AGG AGG ACA GAG TTT ATC GTG	Eurofins genomics
CD68 (Macrosialin)	CCT CGC CTA GTC CAA GGT C	GGA TTC GGA TTT GAA TTT GGG CT	Eurofins genomics
CD11c (Itgax)	CTG GAT AGC CTT TCT TCT GCT G	GCA CAC TGT GTC CGA ACT C	Eurofins genomics
IL-1β	GCA ACT GTT CCT GAA CTC AAC T	TCT TTT GGG GTC CGT CAA CT	Eurofins genomics
TNF-α	CCC CAA AGG GAT GAG AAG TT	CTC CTC CAC TTG GTG GTT TG	Eurofins genomics
MIP-1α (CCL3)	TTC TCT GTA CCA TGA CAC TCT GC	CGT GGA ATC TTC CGG CTG TAG	Eurofins genomics
MCP-1 (CCL2)	CCC CAA GAA GGA ATG GGT CC	GGT TGT GGA AAA GGT AGT GG	Eurofins genomics
IL-6	TCC TAC CCC AAT TTC CAA TGC TC	TTG GAT GGT CTT GGT CCT TAG CC	Eurofins genomics
IL-1Ra	AAA TCT GCT GGG GAC CCT AC	TCT TCT AGT TTG ATA TTT GGT CCT TG	Eurofins genomics
CCR2	GCC ATA CCT GTA AAT GCC ATG C	GGC AGG ATC CAA GCT CCA AT	Eurofins genomics
RANTES	GCT GCT TTG CCT ACC TCT CC	TCG AGT GAC AAA CAC GAC TGC	Eurofins genomics
MMP-9	CAT TCG CGT GGA TAA GGA GT	CAC TGC AGG AGG TCG TAG G	Eurofins genomics
MGL-1	TGA GAA AGG CTT TAA GAA CTG GG	GAC CAC CTG TAG TGA TGT GGG	Eurofins genomics
Arg-1	CTC CAA GCC AAA GTC CTT AGA G	AGG AGC TGT CAT TAG GGA CATC	Eurofins genomics
MRC-2	TAC AGC TCC ACG CTA TGG ATT	CAC TCT CCC AGT TGA GGT ACT	Eurofins genomics
MRC-1	TGA TTA CGA GCA GTG GAA GC	GTT CAC CGT AAG CCC AAT TT	Eurofins genomics
CD8	AAG AAA ATG GAC GCC GAA CTT	AAG CCA TAT AGA CAA CGA AGG TG	Eurofins genomics
iNOS	CAG CTG GGC TGT ACA AAC CTT	CAT TGG AAG TGA AGC GTT TCG	Eurofins genomics
Cyclin D1 (CCND1)	GGG TGG GTT GGA AAT GAA C	TCC TCT CCA AAA TGC CAG AG	Eurofins genomics
Exon 1 PI3Kγ	GCCCCGGGTAGGTCTAGA	GTGATGCGGAGGAGGATCATT	Eurofins genomics
Exon 3 PI3Kγ	TTTGAACCGTACCACGACAGT	CCACGCTTCAGCAGGAATCT	Eurofins genomics

1.5 Biological samples

Description	Source	Identifier

1.6 Deposited data

Name of repository	Identifier	Link

1.7 Software

Software name	Manufacturer	Version
Image Lab	Bio-Rad	6.0.0 build 26
Axiovision	Zeiss	rel 4.6
GraphPad Prism	GraphPad	9.1.2

1.8 Other (e.g. drugs, proteins, vectors etc.)

DEN (N-Nitrosodiethylamine)	SIGMA	Cat # 73861
TUNEL	ROCHE	Cat# 12156792910
AST Assay	Abcam	Cat# ab105135
Triglyceride Assay	Abcam	Cat# ab65336

1.9 Please provide the details of the corresponding methods author for the manuscript:

Giovanni Solinas
Wallenberg Laboratory
Sahlgrenska University Hospital,
Bruna Stråket 16
413 45 Gothenburg, Sweden.
+46 73 5543461
giovanni.solinas@wlab.gu.se

2.0 Please confirm for randomised controlled trials all versions of the clinical protocol are included in the submission. These will be published online as supplementary information.
